# Nurse Managers’ Strategies to Navigate Clinical Leadership and Managerial Responsibilities: A Scoping Review

**DOI:** 10.1155/jonm/2736922

**Published:** 2026-07-20

**Authors:** Subashini Siwan, Rachel Cardwell, Lisa McKenna

**Affiliations:** ^1^ School of Nursing and Midwifery, La Trobe University, Kingsbury Drive, Bundoora 3086, Australia, latrobe.edu.au; ^2^ Health Professions Clinical Education Unit, La Trobe University, Bundoora 3086, Australia, latrobe.edu.au

## Abstract

**Aim:**

To systematically map evidence on how nurse managers (NMs) balance clinical and managerial responsibilities of their role.

**Background:**

Complexity of the NM role arises from its dual focus on both clinical and managerial responsibilities. Clinically, NMs must maintain expertise to guide, support delivery of high‐quality care and ensure care practices align with evidence‐based guidelines and organisational standards. On the managerial side, NMs are responsible for administrative tasks such as budgeting, staffing, performance monitoring and strategic planning. Given their significant impact on healthcare delivery, patient safety and organisational effectiveness, understanding how NMs balance their roles is essential to improving the overall quality of care in healthcare settings.

**Methods:**

Scoping review guided by the Joanna Briggs Institute (JBI) framework for evidence synthesis was conducted. Data were analysed using Braun and Clarke’s (2022) approach.

**Data Sources:**

Studies were identified through seven electronic databases without restrictions on publication dates: CINAHL, Medline, PubMed, Embase, ProQuest Central, Scopus and Web of Science and search engine Google Scholar.

**Reporting Method:**

This scoping review adhered to Preferred Reporting Items for Systematic Reviews and Meta‐Analyses Extension for Scoping Reviews (PRISMA‐ScR) to ensure transparency and rigour.

**Results:**

Forty‐nine studies were included, identifying three main themes: key challenges faced in fulfilling clinical and managerial responsibilities, strategies used to balance roles and factors influencing prioritisation.

**Conclusion:**

Demands of dual roles often cause role ambiguity, conflict and overload, contributing to stress and job dissatisfaction amongst NMs. How they integrate clinical and managerial responsibilities remains underexplored, leaving a gap in understanding processes that guide their prioritisation and decision‐making. Understanding their challenges will help better prepare nurses for their roles.

**Implications for Nursing Management:**

NMs are crucial links between clinical care and organisational management. Further research will inform advancements in nursing leadership, healthcare management and policy development.

## 1. Introduction

Nurse managers (NMs) play pivotal roles in the healthcare system, acting as crucial links between clinical care and organisational management [1]. They are experienced registered nurses (RNs) who transition into leadership roles and are expected to use their clinical knowledge and managerial skills to enhance organisational performance, ensure patient safety and foster positive work environments [2]. Primarily, NMs manage and lead nursing operations within designated service areas in health services [3] and are responsible for ensuring effective coordination and efficient management of these areas or departments. Key functions of clinical leadership and accountability align with the designated health service’s clinical governance and operational management structures [4].

While the title NM is common in many countries, different regions use varying terminology to describe the role. For instance, terms such as nursing unit manager, clinical nurse manager, charge Nurse or ward sister are often used [5, 6]. Regardless of title, essence of the role remains consistent: NMs are responsible for leading and managing nursing staff, ensuring effective patient care and contributing to organisational decision‐making processes [1].

Transition from clinical nurse to manger is significant and often marked by a lack of clear boundaries between clinical and managerial duties [7]. The complex and demanding nature of the role requires them to constantly manage multiple competing priorities [2, 8, 9]. El Haddad et al. [1] assert that NMs have direct impact on quality of patient care, staff performance and overall ward or unit culture. As healthcare systems have become more complex and challenging, the role of NMs has evolved to include expanded leadership and management responsibilities, requiring a balance of clinical and organisational expertise [10, 11]. This multifaceted focus creates unique opportunities and challenges, making the role one of the most complex and demanding positions in healthcare [7].

Historically, NMs were often considered ‘supernumerary’, meaning they were not expected to participate in direct patient care. This structure allowed them to focus on management and leadership tasks without the distraction of clinical duties [2]. However, increasing demand for healthcare services, particularly exacerbated by the COVID‐19 pandemic, has led many to take on direct clinical responsibilities alongside their managerial duties [2, 11]. This shift has intensified challenges associated with balancing clinical and administrative functions, requiring NMs to navigate competing demands and prioritise tasks accordingly [2, 8, 9]. Despite these changes, NMs remain crucial to the success of healthcare organisations [1, 2].

Complexity of the role stems from its dual focus on both clinical and managerial responsibilities. NMs must navigate the unique challenge of shifting seamlessly between being clinical leaders: advocating for patient care, and as organisational managers: responsible for meeting key performance indicators, ensuring regulatory compliance and managing workforce performance and resources [9, 12]. Clinically, NMs must maintain expertise to guide and support nursing teams in delivering high‐quality care. They must ensure care practices align with evidence‐based guidelines and organisational standards. On the managerial side, they are responsible for administrative tasks such as budgeting, staffing, performance monitoring and strategic planning. Demands of both aspects often create role ambiguity, conflict and overload, leading to stress and dissatisfaction [2, 8, 13].

Although the significance of the manager role is well recognised and documented, existing reviews have primarily focused on identifying leadership competencies and managerial skill frameworks for NMs [1, 2, 9, 10, 12]. While these reviews provide valuable insights into core capabilities and responsibilities of the manager role, there is limited understanding of how NMs achieve balancing these responsibilities in practice and factors influencing related decision‐making processes [2, 11, 12]. Many primary studies similarly focus on either clinical or managerial aspects in isolation, without examining the interplay between these roles, leaving a critical gap in understanding how NMs integrate competing responsibilities across diverse organisational and healthcare contexts [9, 14]. Given NMs’ substantial impact on healthcare delivery, patient safety and organisational effectiveness, a focused examination on how NMs balance their roles is essential to inform practice and improve overall quality of care.

## 2. Aim

This scoping review aimed to explore and understand how NMs balance clinical and managerial responsibilities of their roles. It sought to identify key characteristics influencing how NMs balance these roles, as well as other related factors. The primary research question guiding the review was as follows: How do NMs balance clinical and managerial responsibilities?

Secondary questions included the following:

• What strategies do NMs use to balance their dual roles?
• What are the key challenges faced by NMs in fulfilling both clinical and managerial responsibilities?
• What factors influence NMs’ decision‐making in prioritising clinical versus managerial duties?

## 3. Methodology

### 3.1. Design

A scoping review was selected as it enables mapping of existing literature, identification of key concepts, gaps in knowledge and practice and areas for future research [15, 16]. It involves systematic identification and collection of literature, is exploratory in nature and aims to provide a broad understanding of the research question by capturing and presenting the most comprehensive body of evidence possible [17, 18]. This extensive collection of literature provides an overview, helps identify the scope and volume of available research and facilitates analysis and synthesis of evidence related to the research question [19–21].

The review was conducted in accordance with the Joanna Briggs Institute (JBI) methodology for scoping reviews to guide initiation, development and implementation of the study [16]. The Preferred Reporting Items for Systematic Reviews and Meta‐Analyses Extension for Scoping Reviews (PRISMA‐ScR) checklist was followed to ensure adherence to reporting standards [16, 18, 22]. This approach ensured systematic, rigorous, transparent and reproducible findings [21, 22] (Table S1). The JBI mnemonic framework (population, concept and context) informed the inclusion criteria, structured the literature search and provided a strong foundation for the review. This ensured alignment between research title, objectives and eligibility criteria, establishing a clear and focused foundation for the study [21] (Table 1).

**TABLE 1:** JBI PCC framework.

PCC element	Definition
Population	NMs working in any healthcare setting (hospital: private and public, acute and subacute, nursing homes/aged care facilities, long‐term care facilities, community care settings, primary care and rural/remote)
Concept	The dual managerial and clinical components of NMs’ role, including strategies used to balance these responsibilities, challenges encountered, decision‐making processes and available support systems
Context	Open (no restriction on geographical location or healthcare setting)

### 3.2. Eligibility Criteria

The search focused on primary research studies across a broad range of study designs (e.g., qualitative, quantitative and mixed methods) and peer‐reviewed articles in English language. This restriction was applied due to the research team’s primary use of the English language and limited resources. No publication date restrictions were applied to ensure a comprehensive review of all available evidence. Inclusion and exclusion criteria were established through consensus among all authors and reviewed throughout the search process to ensure alignment with primary and secondary research questions, including related concepts. Table 2 outlines the inclusion and exclusion criteria.

**TABLE 2:** Inclusion and exclusion criteria.

Inclusion criteria	Exclusion criteria
Studies on NMs in healthcare settings (hospitals: private and public, acute and subacute, long‐term care, nursing home/aged care facilities, community care settings, primary care, rural)	Studies focussing on clinical or managerial roles without considering the balance between the two
Studies discussing clinical and managerial responsibilities	Non‐English language publications
Studies discussing balance of clinical and managerial responsibilities/duties	Studies not involving NMs or focussing exclusively on other healthcare professionals
Peer‐reviewed articles in English language	Nonprimary research (e.g., reviews, opinion pieces and conference papers)
No publication date restrictions	Studies where full text was not available

### 3.3. Search Strategy

A preliminary broad search was undertaken in two databases to explore the topic, using subject headings and keywords identified while examining the phenomenon of interest. However, this search did not yield relevant studies. Consequently, keywords were refined following team discussions to improve the search’s precision and identify more relevant studies. Assistance from senior research and learning librarians at La Trobe University was sought to design and conduct comprehensive searches. The agreed search terms were applied across seven electronic databases: Medline, PubMed, CINAHL, Scopus, Embase, ProQuest Central and Web of Science Core Collection without time restrictions. Google Scholar was searched using the same terms, with the first 15 pages reviewed to identify additional grey literature. Reference lists of all included studies were screened to ensure key research was identified, followed by a hand search to confirm that no relevant literature was missed [17].

The search included subject headings, keyword synonyms and relevant MeSH terms related to NMs’ dual clinical and managerial roles. Truncations (∗) and Boolean operators (‘AND’ and ‘OR’) were used to capture variations and combine search terms [23]. Search terms included the following: ‘Nurs∗ administrator∗’ OR ‘Nurs∗ leader∗’ OR ‘Nurs∗ supervisor∗’ OR ‘Nurs∗ unit manager∗’ OR ‘Nurs∗ manager’ OR ‘Nurs∗ coordinator∗’ OR ‘Ward manager∗’ OR ‘Ward sister∗’ OR ‘Nurs∗ sister’ AND ‘Clinical responsibilit∗’ OR ‘Manag∗ function∗’ OR ‘Hybrid dut∗’ OR ‘Dual∗ role∗’. The full database search strategy is available in Table S2.

### 3.4. Quality Appraisal

Conducting quality appraisal is important to enhance the final review narrative [21]. Although formal quality appraisal of individual studies is not mandatory in scoping reviews due to their exploratory nature [24, 25], there is ongoing criticism regarding the lack of attention to the quality of studies included in these reviews [25, 26]. Two team members (S.S. and R.C.) independently conducted quality appraisal using the Critical Appraisal Skills Programme (CASP) tools [27–29] to assess the overall quality of evidence. No studies were excluded based on the quality appraisal. The complete quality appraisal is available in Table S3.

### 3.5. Data Extraction and Synthesis

Search results were exported into EndNote 21 [30], grouped and labelled by date and database source. Once all relevant literature was transferred, sourced entries were imported into Covidence [31], a systematic review management tool used to structure data extraction, ensuring consistency and accuracy throughout the review. Covidence enabled the researchers to efficiently screen studies, assess quality, extract data and resolve conflicts in a collaborative and systematic manner [16, 18, 31].

**FIGURE 1:**
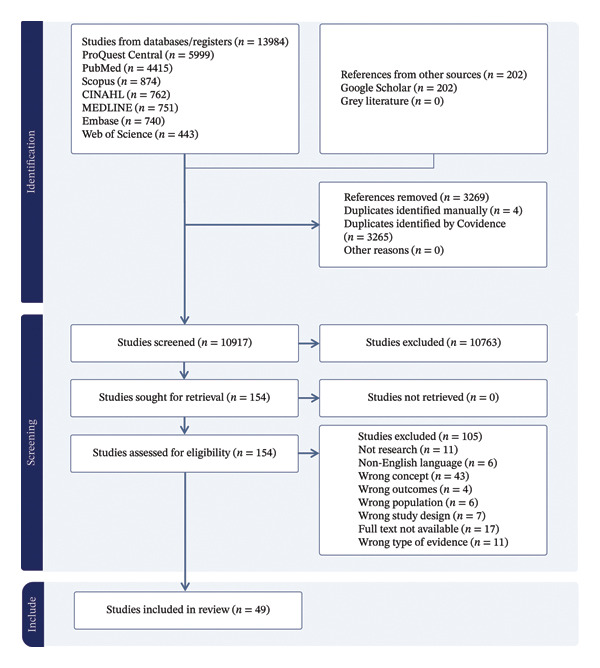
Preferred Reporting Items for Systematic Reviews and Meta‐Analyses (PRISMA) flow diagram [32].

Search results, study selection process and reasons for excluding sources at full‐text stage are documented and presented using the PRISMA flow diagram [32] (Figure 1). Each study was initially screened by two reviewers, who assessed titles and abstracts against the inclusion and exclusion criteria. The same reviewers then conducted full‐text screening, with a third reviewer resolving any conflicts. Finally, data were independently extracted from included studies by two reviewers (L.M. and S.S.) and validated by a third reviewer (R.C.) in Covidence, using a data extraction tool developed by the research team. Data were extracted under the following headings: author(s), year of publication, country of origin, study aim, study population, study design, key findings related to the review questions, gaps and recommendations for future research and study limitations. No modifications were made to the data extraction tool during the process. Any inconsistencies in the extracted data were iteratively discussed and resolved among all three reviewers.

Data were synthesised according to review aims [18] using thematic analysis, as outlined by Braun and Clarke [33, 34], the primary method of data codification [33, 34]. This involved six key steps: (1) familiarisation with the data, (2) generation of initial and preliminary codes, (3) interpretation of codes to develop themes, (4) review and critique of draft themes, (5) confirmation and definition of themes and subthemes, and (6) illustration of themes using extracts for reporting. Thematic analysis identified recurring patterns in the data and enabled the development of a thematic map to visualise relationships between themes and highlight gaps in the literature. This map will guide future research by identifying areas requiring further exploration [33, 34].

## 4. Results

The initial search identified a total of 14,186 studies. Following removal of 3269 duplicates (3265 through Covidence and four manually), 10,917 studies remained for title and abstract screening, of which 10,763 were excluded. Subsequently, 154 full‐text articles were assessed for eligibility. Of these, 105 articles were excluded for the following reasons: nonprimary research (*n* = 11); non‐English‐language publications (*n* = 6); studies not involving NMs or focussing exclusively on other healthcare professionals (*n* = 6); studies focussing solely on clinical or managerial roles without addressing the balance between the two (*n* = 47); wrong study design or evidence type (*n* = 18); and full text not available (*n* = 17). A total of 49 studies were included in the final review (Figure 1).

### 4.1. Characteristics of Included Studies

Table 3 presents characteristics of the included studies. The review included 49 original peer‐reviewed studies published between 1991 and 2024 across 13 countries: Australia (*n* = 10), USA (*n* = 8), the United Kingdom (*n* = 7), Canada (*n* = 6), South Africa (*n* = 5), Finland (*n* = 4), Sweden (*n* = 2), Israel (*n* = 2), and one each from China, Hong Kong, Jordan, New Zealand and Norway. Study designs were classified as qualitative, quantitative or mixed methods based on the methodological approaches described by the original authors, including data collection methods and analytical techniques. Thirty‐nine studies employed qualitative research designs, while five were quantitative and five used mixed methods.

**TABLE 3:** Characteristics of included studies.

Author, year, country	Aim	Study design	Setting	Participants	Findings	Quality appraisal score (%)	Limitations
Algunmeeyn et al., (2023), Jordan	To identify barriers to effective clinical nursing leadership in Jordanian hospitals from perspectives of nurse managers (NMs).	Qualitative. Two focus groups with NMs and two in‐depth interviews with associate executive directors of nursing.	One public and one private hospital in Jordan.	21 (19 NMs and two AEDNs).	‐ Four barriers to clinical nursing leadership emerged: (1) power differential, (2) inconsistent connectedness with physicians, (3) lack of early socialisation experiences and (4) clinical practice reform is a mutual responsibility. ‐ NMs expressed lack of time to role model or mentor.	100%	Limited transferability of findings.

Andersson et al., (2014), Sweden	To explore managers’ experiences of managing everyday work in Swedish emergency departments.	Qualitative. Individual interviews.	Two EDs in Western Sweden.	7 managers comprising 6 nurses and 1 physician.	‐ One theme: ‘Constrained by the systems’ contained three subthemes: Maintaining and securing patients’ vital status while waiting for doctor’s decision, command and control with a circumscribed manager mandate and leading everyday work with difficulty in meeting expectations. ‐ Managers were expected to enable medical emergency, ensure cost efficiency and drive productivity. ‐ Workplace fluctuations caused uncertainty and occasionally chaos, requiring managers to interpret and explain clinical and managerial needs. ‐ Despite needing to be accessible, heavy administrative duties and different working schedules were additional aggravating factors.	90%	Limited transferability. The two EDs were organised in different ways which may have influenced managers’ descriptions.

Bolton, (2003), United Kingdom	To give insights into the role of nurses as managers in the NHS hospital service.	Qualitative. Semistructured interviews and observations.	Gynaecology wards and outpatient clinics in a large North West trust hospital.	Nurses as managers. Sample size not stated.	‐ Nurses distanced themselves from the term ‘manager’ and rejected being viewed in a management role, identifying instead with the image of an altruistically motivated, caring professional. ‐ Roles of nurse and manager were seen as conflicting with their coexistence viewed as unworkable. ‐ Clinical nurse manager (CNM) role was viewed as line management, provoking resentment tied to new management approaches. CNMs frequently had to defend their clinical status and reaffirm their identity as nurses.	55%	Findings not transferable. Methodology, including number of participants, not well described.

Bunsey et al., (1991), United States	To assess relationships among nurse managers’ (NMs) perceived job stress/job satisfaction, time allocation and role expectations.	Cross‐sectional study. Survey comprising: role–conflict and role–ambiguity instrument (Rizzo et al., 1970); First‐line nurse manager tool. NMs job satisfaction measured by Work Satisfaction Scale (WSS) (Atwood & Hinshaw, 1984).	11 community hospitals in North‐Eastern Ohio.	72 NMs.	NMs’ actual and ideal time use largely aligned. Nursing supervisors were satisfied with time allocation of NMs, whereas physicians and nurses felt more of NMs’ time should go to direct patient care. Discrepancies about time allocation of NMs were linked to lower job satisfaction.	86%	Self‐report survey, only one region, only NMs’ perceptions of what nurses, nursing supervisors and physicians thought.

Chisengantambu‐Winters et al., (2024), Australia	To develop greater understanding of roles and tasks performed by rural nurse managers (RNMs), drawing on nurses’ own experiences and understandings.	Qualitative. Face‐to‐face semistructured in‐depth interviews, direct participant observation.	Nonmetropolitan healthcare facilities in South Australia, in communities classified as Regional (Rural) or Remote /Very Remote (Australian Statistical Geography Standard (ASGS) Edition 3).	15 (11 rural and 4 remote nurse managers).	Three themes: ‐ Varied roles and responsibilities: RNMs prioritised hands‐on care and staff support, viewing clinical work as central, even if not reflected in workload. Alongside managerial responsibilities, they acted as care providers, skill mentors and liaisons across health sectors and departments. ‐ High workload, stress and dissatisfaction: Unlike their metropolitan counterparts who shared tasks, RNMs especially in remote areas often handled all responsibilities alone with fewer RNMs. This broad workload, combined with lack of support, insufficient managerial training and ageing RNM workforce, contributed to ineffective performance, stress and burnout and absenteeism. ‐ Role expansion: RNMs had broader duties than urban counterparts, adapting to rurality, professional isolation and service gaps. Their roles evolved from clinical to diverse managerial tasks. Described as ‘jack‐of‐all‐trades’, many worked extended hours due to unplanned duties and unmet demands driven by social and political pressures.	90%	RNMs at only 15 healthcare facilities in rural and remote South Australia at one point in time. Findings not transferable.

Cilliers & Terblanche, (2010), South Africa	To describe systems psychodynamic learning experiences of nursing managers during leadership coaching.	Qualitative. 10 x1 hour weekly coaching sessions.	Hospitals in one private hospital group in Gauteng.	9 senior nursing managers.	‐ Anxiety: Recognised how anxiety influenced their leadership roles. ‐ Task: Preferred clear mechanical and tasks over complex interpersonal dynamics with staff and peers. ‐ Role: Found comfort in rational aspects of their role, avoiding emotional complexities like projections and introjections. ‐ Authorisation: Felt consciously authorised from above, side and below in normative role but unconsciously deauthorised by those in power, management and leadership positions. ‐ Boundaries: Became aware of the need for clear interpersonal and professional boundaries to strengthen their nurse manager role. ‐ Identity: Had clarity in job performance but felt burdened by the unspoken expectations to absorb systemic anxiety.	95%	Limited transferability, participants from one private hospital group.

Cziraki et al., (2014), Canada	To determine factors that attract and retain registered nurses (RNs) in the first‐line nurse manager (FLNM) role.	Qualitative. Individual semistructured interviews.	A large, regional healthcare organisation with five sites, 1200 beds and 10,000 employees in Central South Ontario, Canada.	11 RNs in FLNM roles from medical, surgical, critical care and ambulatory care settings.	‐ Factors attracting: Meaningful work; Factors retaining: Passion, pride and continuing to grow. ‐ Challenges must be addressed to attract and retain RNs in FLNM roles. ‐ Collaboration and respect: Conflicts between FLNM and union representatives due to delays in hiring process. ‐ Managing complexity: Role expanded from unit‐based nursing to overseeing diverse professional teams and financial/regional tasks. ‐ Organisation and program support: Less than half reported having adequate support, unmet expectations including unavailable mentors and limited influence on change. ‐ Workload and scope: Disparity between demands of nurse manager role and hours of work. ‐ Rewards: Salary discrepancies with other specialities required review.	90%	Two study sites only. Limited transferability. Convenience sampling.

Dienemann & Shaffer, (1992), United States	To assess responsibilities and activities of nursing unit managers (NUMs) in hospital and community health settings.	Mixed methods. Semistructured interviews with Chief Nurse Executives and survey with NUMs.	Six hospitals and two county health departments in mid‐Atlantic metropolitan area.	73 (48 from six hospitals and 25 from two county health departments).	‐ Nursing service and human resources accounted for more than half of NUMs’ time, 27% in each category. ‐ 12% of time allocated to direct patient care. ‐ Community health NUMs spent 34.48% in HR management, compared to 23.06% for hospital NUMs.	73.5%	Small study, qualitative findings not presented. Findings not generalisable.

Drach‐Zahavy & Dagan, (2002), Israel	To depict essence of what head nurses do, and how they perform their managerial role.	Observational. Semistructured observation. Each nurse observed for average of 6 h in 2 sessions on 2 different days and 2 different times of day.	Departments in various hospitals representing surgical, internal medicine, obstetrics, gynaecology and neonatal.	48 head nurses.	‐ 29 functions observed across 7 categories: clinical care, system coordination, operations, leadership, personnel management, quality improvement and other. ‐ Head nurses spent almost 41% of time in primary nursing of patients, and with families in conversation, instruction and consultation. They engaged mainly in coordinating interprofessional team face‐to‐face (7.33%) and committee work (7.12%). ‐ ‘Operations’: 3rd largest category of functions, 14.8% of time. ‐ ‘Leadership’ 4th largest among functions: about 10% of time spent on development of nurses individually and smooth team functioning. ‐ Remaining categories; negligible proportions of time: personnel management (3.5%), quality improvement (2.15%), other (7.68%). Marginal share of time spent outside unit: 6.40% in other units, 2% outside hospital, and 1% in administration offices. ‐ Less senior head nurses and those in smaller departments spent more time on leadership and less on quality, management and coordinating than head nurses of large departments.	70%	Potential for observer bias. Only snapshots of activities were obtained. Some activities may not have been observed or observable.

Duffield et al., (2019), Australia	To explore extent of Australian nurse managers’ (NMs) engagement in clinical care activities.	Cross‐sectional survey comprising Advanced Practice Role Delineation (APRD) tool (Gardner et al., 2016)‐41 activity items across 5 domains of clinical care, support of systems, education, research and professional leadership.	National survey of Australian registered nurses (RNs).	2,758 RNs nurses, 390 clinical (front‐line) NMs and 43 organisational (middle) NMs.	‐ Clinical NMs in hybrid roles reported higher levels of engagement across domains than organisational NMs, who focussed more on strategic tasks as they advanced in hierarchy. ‐ Clinical NMs scored higher in all domains, except clinical care, where RNs rated significantly higher. Clinical NMs had a median engagement score of 2.1 in clinical care, compared to 1.5 for organisational NMs, who were engaged but less so. ‐ Both roles supported clinical care, but clinical NMs were more involved in front‐line activities. Their higher scores in the support of systems domain reflected involvement in both strategic and direct care improvement efforts.	100%	APRD tool does not adequately capture NMs’ other managerial and leadership activities.

El Haddad et al., (2022), Australia	To capture and explore nursing unit managers’ (NUMs) perceptions of their work and leadership practices within a large, multifacility tertiary healthcare organisation.	Cross‐sectional survey comprising combination of validated and purpose developed scales, and open‐ended questions. Validated scales: Utrecht Work Engagement Scale (Schaufeli & Bakker, 2004), Survey of Perceived Organisational Support (University of Delaware, 1984), and modified version of Intention to Stay Scale (Shacklock & Brunetto, 2012).	A large and diverse public regional healthcare organisation in Queensland.	34 NUMs.	‐ NUMs reported working average of 25 h each per week on administrative activities, corresponding to 0.6 FTE. Activities contributing most to administration time were payroll and roster management; with monthly reports, clerical work, recruitment, mandatory training and stock inventory taking less. ‐ They reported enacting all leadership capabilities (presented in averaged scores) of Clinical Leadership Profile (CLP). Relative to average scores, most enacted capability was ‘engages others’, ‘drives innovation’ and ‘achieves outcomes’. Least enacted capabilities were ‘leads self’ (3.6) and ‘shapes systems’. ‐ Work engagement was driven by high dedication to absorption constructs, with vigour rated lower. Those with postregistration qualifications showed slightly higher dedication and absorption than those with only preregistration qualifications. ‐ Perceived organisational support was met with ambivalence, through NUMs with < 5 years in role were slightly more positive about organisational support than those with more experience.	100%	Small convenience sample from a single organisation, self‐reported.

Ericsson & Augustinsson, (2015), Sweden	To describe ward managers’ (WMs) experiences of their professional role, their work and how they were handling their everyday practice.	Multiple methods. Participants followed for approximately 4 years. Data collected through interviews, observations and continuous dialogue forum (3 years).	One Swedish hospital.	5 WMs.	Four themes: ‐ From supervisor to manager: Participants identified more as managers than nurses lacking clinical skills and competence in nursing work. They felt responsible for representing the ward externally and relaying information to staff. ‐ Loyalty: They expressed ambivalence about their role, in terms of who and what they represented. Formally, the WM/first‐line manager (FLM) was part of the management system and loyal to management demands. They perceived their role as torn between management expectations and advocacy for nurses and assistant nurses. Many felt excluded from the management system and spent less time on the ward due to frequent meetings. ‐ Talking about it: Administrative tasks and staffing dominated their work. While they aimed to lead beyond everyday schedule, development work was often sidelined. Their role involved informing and clarifying organisational matters for staff. ‐ Dialogue forum: WMs described frustration with limited resources and support, describing their role as navigating the organisation and contributing to staff in constructive ways.	80%	Findings not transferable, one hospital in one country.

Gaskin et al., (2012), Australia	To investigate challenges nurse unit managers (NUMs) face while working in acute care settings, strategies they use to deal with these challenges and effectiveness of strategies from perspectives of NUMs and their supervisor.	Qualitative. Semistructured interviews.	Five acute care settings in Melbourne, Australia.	22 NUMs, 3 directors of nursing (DONs).	14 challenges identified relating to NUMs’ interactions with others, both in and outside of their wards/units. ‐ Preparedness for NUM position: Insufficient skills and limited support. ‐ Personal issues: NUMs sometimes sought counsel for issues from DONs. ‐ Scheduling: Heavy workloads; competing clinical and managerial demands and management time pressures were common; 77% had approx. 50% clinical loads, with clinical needs often taking priority. ‐ Staff management: Building cohesive teams and resolving staff issues. ‐ Resource management: 3 strong subthemes (i.e., insufficient equipment, staffing disparity across hospital sites, insufficient multidisciplinary and support staff) and 1 subtheme mentioned by only one (i.e., limited financial information). ‐ Patient issues: DONs identified managing large patient numbers was challenging. 4 subthemes covering a variety of patient issues: Addressing concerns of patients and visitors; dealing with difficult patients and visitors; increased nurse workload; and patient acuity. ‐ Conflict management: NUMs mediated staff conflicts and were sometimes directly involved. ‐ Organisational demands: NUMs worked in a complex matrix system, responsible to program (e.g., medical and surgical) and site (i.e., multiple hospitals within the network) supervisors. ‐ Change management: DONs stated NUMs often did not receive extra resources to enable implementing requested changes. For NUMs, 2 main subthemes: Lack of influence to implement change and minimal consultation. ‐ Multidisciplinary coordination: Various staff contributed to patient care, directly influencing effectiveness and efficiency of units but NUMs had no authority over these staff. ‐ Range of support from organisation: NUMs indicated not always receiving assistance for problems encountered (e.g., staffing issues and administrative issues). Sometimes needed advice on addressing staffing issues and did not receive sufficient emotional support when experiencing high workloads. ‐ Managing high expectations of others: NUMs experienced difficulties managing others’ expectations, including their supervisors (i.e., DONs). NUMs perceived expectations sometimes unmanageable and impractical. ‐ Poor interunit communication: Misunderstanding capabilities of unit staff and problems moving patients between units. ‐ Information systems: Time to complete electronic forms, using databases that were not linked, performing tasks manually as no electronic systems in place, receiving inadequate training in some electronic systems and limited practical support from information technology staff. 16 strategies for managing challenges identified: seeking assistance and support; trial and error; satisficing; taking responsibility for own professional development; scheduling time; working longer hours; delegation; adaptive staffing and rostering; being a visible presence on the ward; team development; facilitating professional development for staff; being available for staff; negotiation and collaboration; communication; working with processes of a large organisation; and complying with others’ demands.	90%	Staff from one health service in one geographical location only. Only 3 DONs participated.

Gould, (2008), United Kingdom	To describe how matrons in an acute National Health Service (NHS) trust perceive and undertake their role since its reconfiguration in 2005 and to investigate their needs for continuing professional development.	Qualitative. Interviews.	One acute NHS trust.	22 matrons.	‐ Patients, families and staff welfare took priority, with meetings and administration fitted around them. Most relied on clinical experiences as ward sisters to support staff and uphold patient care standards. ‐ A typical day involved visiting clinical areas to support patients and staff and address urgent issues. Matrons valued the freedom to engage with patients and provide direct care. Some supported students, guided junior staff or conducted matron’s rounds, blending clinical duties with high visibility seen as key to the role. ‐ Most felt overburdened by nonclinical activities, especially human resource issues, and perceived they were expected to perform tasks beyond their role or avoided by others. Many reduced meeting attendance, often in creative ways. ‐ Frustrations included the rapid pace of NHS change, pressure to meet targets, financial pressures and lack of budget control, often managed by nonclinical general managers.	90%	One health trust only. Limited transferability.

Ingersoll et al., (1999), United States	To determine effect of patient‐focused redesign on mid‐level nurse managers’ (NMs) role, responsibilities and perceptions of work environment.	Qualitative. Interviews.	2 tertiary care hospitals in the Mid‐West, both referral centres.	9 mid‐level NMs.	Themes: ‐ Expanded role responsibilities: NMs need to measure and manage outcomes, understand organisational finances and external pressures, build strong teams and manage rapid change. Mid‐level managers were expected to lead in demanding, high‐stress roles. Despite supporting and respecting senior executives, many felt isolated and disconnected from both superiors and subordinates. They doubted their ability to lead disrupted teams and feared misalignment with executive decisions. Inability to resolve staff issues or answer questions left many feeling frustrated and inadequate. ‐ Decreased self‐esteem: Over time, NMs doubted their leadership abilities, struggling to meet expectations. Previously effective strategies no longer worked, and they felt unable to provide emotional support to all who needed it. Daily disruptions overwhelmed them, and past experience offered little guidance. They worried about staff nurses’ grief and loss, and the impact of ongoing turmoil on patients and families. Raised loss of credibility and need to reestablish trust. While negative responses were not directed at them personally, constant exposure to conflict made it difficult to stay positive and supportive. Some mid‐level managers questioned their control over current and planned changes, citing limited involvement in early redesign planning contributed to uncertainty about the overall vision and hindered their ability to communicate effectively with staff and physicians. Although they assumed greater responsibility within the organisational structure, many were unclear about how their roles fit into broader decision‐making processes. ‐ Environmental uncertainty: Struggled to balance their roles as patient/staff advocates and agents of organisational change. They faced hostile and angry staff, who often blamed them for their emotional turmoil. ‐ Care delivery environment: Mid‐level NMs addressed concerns from families about care quality and staff, physicians amid workforce changes and departmental shifts as they perceived loss of control of patients. Role confusion and need for redefined responsibilities led to frustration. Despite feeling inadequate at times, NMs employed specific strategies to assess and respond to evolving needs.	80%	Limited transferability, two hospitals in one locality.

Kagan et al., (2021), Israel	To examine managerial and clinical challenges of nurse managers (NMs) in mental health centres during the COVID‐19 pandemic.	Mixed methods. Questionnaire examined NMs’ (a) background data, (b) communication with staff nurses, (c) perceptions of nurses’ functioning, (d) perceptions of own functioning and (e) management as impacted by the pandemic. Three focus groups sessions—NMs discussed challenging and positive issues during the pandemic.	Two mental health centres in Israel.	25 mental health NMs.	‐ Questionnaire: Most important challenges reported were related to clinical impact of the pandemic: Need to protect patients from infection; communication with families and primary caregivers; and impaired communication between patients and their family and relatives. ‐ NMs’ professional functioning on the ward showed both negative and positive consequences of working during the pandemic. NMs reported work policies and procedures were less adapted for the pandemic; advanced work and project execution were decreased; and working relationships with multidisciplinary team weakened. However, they felt sense of purpose, duty and pride and were more disciplined compared to prepandemic times. NMs felt burned out and overwhelmed by new tasks and duties but demonstrated high satisfaction with their managerial performance. ‐ Focus groups, three themes: ‐ Management complexity: Routine job completely changed with the pandemic. To reduce risk of exposure and infection, worked in capsule formations and complex to determine capsules. NMs reported change in professional roles, functioning as ordinary nurses not managers. Nurses expressed sense of loneliness in management. ‐ Challenging communication: Communication with team via software and phones, with limited personal encounter. NMs found it difficult to manage staff. ‐ Bright spots: NMs favourably noted uniformity of staff, sense of cohesion and shared responsibility. Close relationships with patients developed due to the situation.	78.5%	Limited transferability, only mental health departments. Only reflected NMs’ perspectives.

Keutchafo & Kerr, (2019), South Africa	To describe difficulties, in day‐to‐day activities, of unit managers (UMs) in selected Cameroonian district hospitals.	Qualitative. Semistructured interviews.	Two district hospitals in Yaounde, Cameroon.	10 UMs.	Overall theme: ‘It is not easy’ Subtheme 1: Facing difficulties: Expressed difficulties faced daily as UMs, some patient‐related. ‐ In Cameroon, there is no medical aid scheme, and patients pay for everything in public hospitals. UMs had difficulty attending to patients with limited finances. They expressed having trouble leading staff, some mentioning nurses did not listen or comply with instructions. Subtheme 2: Making sacrifices: UMs made sacrifices to cope with professional responsibilities. For male UMs, foregoing other income generating activities. For females, having to make additional arrangements for their children’s care as they had to stay longer at the hospital to ensure everything was done. ‐ UMs mentioned sacrifices made for good functioning of the service like buying supplies with their own money. Subtheme 3: ‘It is not worth it’: After facing difficulties and making sacrifices, UMs expected reward. Agreed financial motivation for ward charges in district hospital insufficient compared to what was required of them. Subtheme 4: Assistance from others: Strategies to overcome difficulties were to ask for assistance from others, head nurse, organisation, family members or from God.	100%	Limited transferability. Potential self‐report bias.

Kirchhoff & Karlsson, (2018), Norway	To explore influence of social support on nurse managers’ (NMs) responses to role conflict.	Qualitative. Participant observation and interviews.	Seven organisational units in Norway.	7 first‐line nurse managers (FLNMs): 4 in 2 municipalities and 3 in hospital wards.	‐ All FLNMs acknowledged organisational change emphasised managerial roles (responsibility and accountability) to execute and implement directives from superiors, including all experienced decentralisation of authority to their level of management, high number of managerial tasks related to this change in authority and frequent interactions with superiors and other FLNMS. ‐ Those working in municipalities received demands from superiors to reduce expenditure, rendering budget control and financial accountability as essential parts of their manager role. In hospital, superiors expected FLNMs to reorganise hospital routines and implement technology in line with changes directed by senior management. ‐ Six FLNMs reported role conflict between their managerial and RN roles as they maintained affiliation. One denied role conflict as she renounced her previous RN role. Two strategies FLNMs adopted due to organisational, and employee demands and incidences of role conflict, categorised as ‘emphasising managerialism career’ and ‘emphasising professionalism career’ to balance roles as nurses and managers. ‐ Role distance careers: Emphasising managerialism career. FLNMs choosing managerial role, distanced them from RN role through loyalty to organisational roles and demands, avoiding nursing activities in the unit and reluctance to participate in debates about specific patient care. Role distance required FLNMs to hold RNs accountable for providing services in line with professional standards. ‐ Emphasising professionalism career: FLNMs identified primarily as RNs and established role distance from managerial role. A common feature was effort to communicate their affiliation with RN role when interacting with employees. FLNMs rarely signalled or highlighted their manager role when interacting with employees, thus distancing themselves from that role. Role distance from managerial role was less comprehensive and visible to employees. ‐ FLNMs who emphasised professionalism allocated resources in line with employees’ demands and sometimes deviated from organisational demands. ‐ A managerial position granted FLNMs authority to supervise and encourage employees to provide services in line with standards of the nursing profession. FLNMs who used this strategy tried to be visible and display interest in meeting with their employees. By engaging in frequent interactions with employees, FLNMs supported and confirmed their roles as nurses. Organisational change often resulted in increased managerial tasks and management meetings. Hence, being prevented from interacting with employees and receiving social support in the unit.	90%	Findings not transferable. Small sample size, data saturation not achieved.

Luo et al., (2016), China	To identify core competencies needed in transition of nurse managers (NMs) on the way to excellence.	Qualitative. In‐depth interviews.	Six hospitals affiliated with the Shanghai Jiao Tong University School of Medicine.	12 NMs with more than three years’ experience.	Four main themes: ‐ Adaptive phase: Upon becoming team leaders, NMs immediately demonstrated high organisational commitment. They prioritised strengthening clinical knowledge to gain credibility and overcome scepticism from staff. As they settled into their roles, they developed professional credibility through role modelling, while becoming aware of the complexities of management. With limited prior management knowledge, many struggled initially with tasks like rostering and financial management. They developed foundational management skills through trial and error, guided by immediate clinical demands that enhanced their operational awareness. ‐ Running‐in and stable phase: NMs began to developing abilities in task organisation and time management. Sometimes, they bore full responsibility for unit‐level decision‐making, including policy and workflow revisions. Six preferred immediate action, and four used negotiation to gain staff support. Some highlighted empowerment to improving work efficiency. Ensuring high‐quality patient care remained central to their role. Fairness was the most important principle when evaluating staff, and effective communication was seen as a vital competency, given the need to liaise across teams and services. Four NMs sought collaboration with chief physicians to resolve clinical issues. With experience, they engaged in self‐reflection to assess their strengths and weaknesses. ‐ Stagnation phase: Three struggled with emotions and became easily depressed. When discussing work–life balance, 10 became emotional and cried. Three older NMs expressed losing motivation and lacked career planning. ‐ Maturation phase: After overcoming the learning ‘hump’, NMs improved steadily and reassessed their management competencies. Eight began developing professional identity and tried to learn second languages to engage in in international exchanges and bridge gaps between domestic and global nursing practices. Rather than focussing solely on routine tasks, they cultivated conceptual and analytical thinking skills essential for risk management. Four reported the ability to identify key risk elements, analyse related factors, and apply scientific methods to resolve issues. Most focused on building their own talent team.	90%	Limited transferability, one province in China.

Maguire et al., (2023), Australia	To explore the role of nurse unit managers (NUMs) working within a forensic mental health service in Victoria, Australia.	Qualitative. Five focus groups.	One hospital in Melbourne.	5 NUMs, 16 health providers working with, or managing, NUMs and 10 health providers managed by NUMs.	Four themes: ‐ Lack of role clarity: NUM role was extensive, covering wide range of duties. Unclear role boundaries often led to taking on responsibilities beyond their remit. Many felt they were expected to be ‘everything to everybody’, with an expectation to manage all aspects of the unit. This ambiguity made it difficult for others to understand what fell within the NUM’s scope. As a result, NUMs frequently worked beyond paid hours. ‐ Importance of clinical leadership and forensic mental health knowledge: Expectation NUMs should function in clinical and managerial leadership roles, managing unit in all aspects including giving feedback, uniting the team together and decisions resulting in positive impact. Ability to manage change and provide mentorship to the team especially when they encountered difficulties. ‐ Step up in responsibility and step down in pay: Gap existed between the high expectations of NUM role and remuneration offered, acting as a disincentive to take on the role. While the role was very important within the organisation particularly in driving change, setting culture, ensuring quality of care for consumers and nurturing and supporting staff, key indicators of its value were lacking, particularly in arrangements for backfilling NMs during leave or absence. ‐ Seeing the difference you make: Despite stress and demands of the role, positive aspects motivated NUMs to remain, including opportunity to make a difference in the lives of consumers and families, ongoing clinical involvement and mentoring junior and new staff. Having direct consumer contact and contributing to care outcomes were highly valued. Managing diverse clinical staff, NUMs saw their role as uniquely positioned to support staff development and positively impact care.	100%	Limited transferability, one forensic mental health service. No medical staff participated.

McCallin & Frankson, (2010), New Zealand	To explore the charge nurse manager (NM) role.	Qualitative. Face‐to‐face structured interview.	One acute care hospital in New Zealand.	12 NMs.	3 themes: Role ambiguity, Business management deficit and Role overload. ‐ Role was diverse compared with job description. Management skills required different to clinical skills charge nurses had on entry to the role. Integrating nursing expertise with management responsibility was challenging. ‐ Participants found quickly they did not have human resource management skills needed to manage staff. ‐ Multiple demands from wide ranging sources such as staff, colleagues, patients, families and wider organisation led to feeling role expectations caused role overload. High expectations challenged charge NMs, asked to do too many things, for too many people, in too little time. They felt unable to meet everyone’s needs, and when support was lacking, their management was questioned.	90%	Participants drawn from one organisation only. Limited transferability.

McEwen et al., (2005), United Kingdom	To investigate self‐reported duties carried out by sisters and charge nurses working on wards and to assess attitudes of these healthcare professionals.	Cross‐sectional study. 34‐item questionnaire designed to identify sisters’/charge nurses’ views about carrying out their duties, piloted within the nursing research team.	St George’s Healthcare NHS Trust in London.	45 ward sisters and charge nurses.	‐ Sisters/charge nurses reported assessing on average 75% patients on their ward during a typical shift. They were allocated patients they had not planned to take primary responsibility for, in addition to being in charge of the ward a mean of 2.5 shifts/wk. They reported spending almost 6 h/wk outside of contracted hours on ward business. More than 2/3 had insufficient time to teach, assess and develop junior staff. 11% had sufficient time to complete all managerial duties each shift and only one‐third agreed they had necessary skills, training, experience and time to manage their teams effectively. ‐ 54% felt senior nurses and trust managers fully supported them, although evenly split on whether trust valued their role. 58% had clear future vision for their clinical area, and same percentage had authority and support to make changes in their area. ‐ 1/3 disagreed that their role allowed them to maintain ongoing personal and professional development. 33% agreed that their skills and experience were not fully utilised in their role. ‐ Over half lacked time or opportunity for regular clinical supervision, though nearly half participated in peer networks to share support and innovations.	73%	Not generalisable. Self‐reported. Small sample size.

Moore et al., (2016), United States	To understand insights of nurse managers (NMs) regarding the nurse manager (NM) role.	Qualitative. Interviews.	Five large, urban Midwestern US acute care organisations.	13 nurses holding title of NM or similar in at least one department or nursing unit within an acute care facility.	‐ Four overarching topics: Path to becoming a NM, what they ‘wished for’ as managers to help them grow, personal attributes that contributed to their success and lessons learnt. ‐ 9 participants identified hunger for more formal mentorship or coaches to ‘walk alongside’, guiding and supporting them. Lack of mentorship for those new to the role and those with experience. ‐ One noted, for novice managers, ‘assigned’ mentors would be helpful to ‘go to with all the questions.’ Others noted bosses could serve as mentors, but time constraints often got in the way. ‐ 7 spoke of learning ‘the art of managing role demands.’ Some demands came with role implementation, while others were more personal such as recognition and maintenance of boundaries. ‐ One addressed discomfort of being between staff and organisation when decisions were being made. 7 participants discussed adjusting to the role did not happen immediately.	100%	Limited transferability and geographical setting.

Nagle et al., (2021), Australia	To explore understanding and experience of nurse and midwifery unit managers (NMUMs) regarding their role; to explore what barriers and facilitators NMUMs identified to achieving goals of their clinical area; and explore NMUMs’ career plans.	Qualitative. Semistructured face‐to‐face interviews.	Four metropolitan hospitals from one health service in Victoria, Australia.	39 NMUMs.	Two overarching themes: System challenges and influences on people; each had three subthemes. ‐ Managerial roles expanded over time, encompassing patient care, staff leadership, administration and resource management. Structural challenges: institutional deficits, inadequate financial and physical infrastructure made the role more burdensome. Many were unprepared for the NMUM role and received limited support. Concerned that administrative duties often reduced clinical time. Key functions and challenges included staffing, skill mix issues and managing difficult staff. Few were able to delegate aspects of their role. ‐ Being an accessible leader was very important, with leadership demonstrated by leading by example and taking responsibility to influence staff behaviour. Effective leadership involved building functional teams that achieved goals through complementary skill sets. Clinical responsibilities were central, with NMUMs working ‘on the floor’ to ensure high standards of care. Despite constraints, patient‐first care was a core mission. Multidisciplinary collaboration through communication and cooperation was crucial to uphold patient interests. ‐ NMUM role was described in terms of personal sacrifices, routinely working long hours to ensure positive patient outcomes and unit success, often at the expense of family life.	90%	Participants from four hospitals in one health service limits transferability.

Narinen & Kekki, (2003), Finland	‐ To study nurse managers’ (NMs) work and analyse different factors related to variation in content. ‐ To explore NMs’ views of how education provided appropriate knowledge and skills, opinions of their own competency and preparedness in relation to content of their work, and analyse factors related to experienced competency. ‐ To examine NMs’ opinions of how they had been supported, and analyse factors related to this. To compare opinions of NMs and DON in terms of content of NMs’ work and experienced competency in relation to content of the work.	Cross‐sectional study. Questionnaire: demographic data (14 items), statements about how NMs had been supported (9 items), content of work (84 items), related items about how NM’s education had provided knowledge for work‐related tasks (20 items) and how NMs assessed their own competency and preparedness for their present tasks (5 items). Last part questions about required skills (36 items).	10 hospitals from 17 different hospital districts in Finland.	17 NUMs on the Delphi panel, and 1812 NMs and DONs participated in the study.	‐ NMs were involved in clinical nursing for almost half of their working hours. Amount of clinical work was determined by the unit work model (OR = 0.03, *p* > 0.05). When NMs participated in clinical work as equal members of the team, about 66% of their working hours were spent in clinical work. If NMs only took care of specific tasks (OR = 0.01, *p* < 0.05), clinical work accounted for 46% and NMs were only consulted when necessary or if some other work model was applied (OR = 1, reference group), clinical work only accounted for 20% of NM’s total work hours. ‐ Taking care of staff administration varied by tasks (recruiting, salaries and wages, decisions about annual holidays and granting leave). Budgeting responsibilities also varied by tasks (budget planning, follow‐up, accountability, acquisition, invoicing and calculation of costs). ‐ Most felt supervisory tasks in units were systematically organised (88% were of this opinion as to staff meetings, 83% as to expanding and enriching scope of work, and 59% as to giving feedback). NMs also felt they had a lot of collaborative and developmental tasks (76% mentioned internal cooperation, 30% external cooperation, 64% development of patient care, and 48% project work). One‐third believed they were poorly supported by superiors.	91%	This was one part of a Delphi study.

Nene, (2022), South Africa	To understand nurse managers’ (NMs) leadership roles in mining primary healthcare settings (mPHCs).	Qualitative. In‐depth semistructured interviews.	mPHCs in Gauteng province in the West Rand.	10 NMs.	Three themes: ‐ Confusion of leadership roles with management roles: NMs unaware that their responsibilities aligned more with general management than leadership. ‐ Confusion of leadership roles with clinical roles: NMs oversaw clinical roles and were expected to step in when needed, such as treating mine workers. They equated leadership with clinical responsibilities, including assessment, treatment, care and developing programs like TB management when required. ‐ Confusion of leadership roles with resources management: NMs identify resource management as their leadership role.	100%	Participants only sourced from one province. Limited transferability.

Nene et al., (2020), South Africa	To explore and describe nurse managers’ (NMs) experiences of their leadership roles in a specific mining primary healthcare service (mPHCs) on the West Rand, to develop recommendations to enhance these roles.	Qualitative. In‐depth interviews.	A specific mPHC service at the West Rand.	7 NMs.	‐ Leadership role ambiguity: All NMs demonstrated leadership role ambiguity, often confusing it with managerial, clinical roles and resource management. They described daily activities as leadership with unclear expectations and guidance contributing to the confusion. ‐ Leadership roles experienced: NMs experienced some leadership roles during their practice. These were described as being coordinators and facilitators of processes, engaging with stakeholders, supporting teams and empowering personnel. One leadership role was stakeholder engagement and team support. ‐ Challenges experienced in leadership roles: lack of effective communication, bureaucratic mining management and union influence. They experienced communication problems, creating a gap affecting PHC service delivery. They experienced bureaucratic directives from mining management, and this impeded execution of their leadership roles. NMs commented that union influence was too strong and challenged their roles.	100%	Limited transferability, one service in one geographical area.

Nurmeksela et al., (2021), Finland	To describe relationships between nurse managers’ (NMs) work activities, nurses’ job satisfaction, patient satisfaction and medication errors at the hospital unit level.	Cross‐sectional study. Questionnaire comprising demographic characteristics, Nurse Managers’ Work Content Questionnaire (NMWCQ), Kuopio University Hospital Job Satisfaction Scale (KUHJSS), Revised Humane Caring Scale and register data of medication errors.	28 units across three Finnish acute care hospitals.	29 NMs, 306 nurses, and 651 patients.	‐ Clinical working being least frequently performed activity and organising being most frequently performed. ‐ Nurses’ total job satisfaction was 7.36 (scale of 0–10), with requiring factors of work and motivating factors of work subscales receiving lowest and highest mean scores, respectively. ‐ Mean score for total patient satisfaction was 8.74, with human resources and professional practice subscales showing lowest and highest scores, respectively. ‐ Nurses’ assessments of general factors of their work were rather poor even though NMs were frequently involved in staff orientation and solving patient complaints. Frequency in which NMs performed work well‐being duties and number of medication errors were both found to decrease patient perceptions of interdisciplinary collaboration. Frequency at which NMs participated in development of nursing duties and nurses’ ratings of requiring factors of work were both negatively related to patient perceptions of Cognition of physical needs. Requiring factors of work subarea of nurses’ job satisfaction, total patient satisfaction and medication errors identified as variables most significantly affected by other factors.	100%	‐ Only 28 units met inclusion criteria. ‐ Small sample of NMs. ‐ Only studied patient satisfaction, nurses’ job satisfaction and medication errors at the unit level. ‐ NMWCQ is a new instrument and, as such, needs to be tested more. ‐ All questionnaires based on self‐report. ‐ Medication error data are based on nurses’ initiative to report.

Paliadelis, (2008), Australia	To explore responsibility and power of the role of nursing unit managers (NUMs) in rural New South Wales using Kanter’s theory of organisational power as a framework.	Qualitative. In‐depth individual interviews.	Health service in the New England area of New South Wales, Australia.	20 NUMs	‐ NUMs ill‐prepared and overwhelmed by scope of the role and limited access to sufficient information and resources, in particular, time, staff and money. ‐ Level 1 NUMs experienced significant tension balancing dual clinical and managerial roles. The competing demands made the role overwhelming and challenging to manage effectively. ‐ NUM described the expectations of their role, highlighted lack of information and resources created tension between managerial and clinical responsibilities. One noted the conflict extended beyond time constraints, challenging their core nursing values. ‐ Transition from Level 1 to Level 3 NUM was challenging, feeling unprepared for the role and meant giving up clinical component of the Level 1 role. ‐ Described the role defined by multiple responsibilities with lack of power to control things such as budget.	100%	Limited transferability, potential response bias, small sample size, single organisation.

Paliadelis, (2005), Australia	To explore nurse unit managers’ (NUMs) stories about the education and support they received in their role.	Qualitative. Semistructured interviews.	One regional health authority in northern New South Wales, Australia (20 public hospitals and community health centres).	20 NUMs	‐ Learning to be a NUM: Overwhelmed, underprepared and unsupported, by new role, when taking on and after. ‐ Education and training: Majority lacked formal managerial qualifications, despite health management credentials or a willingness to pursue them being stated as essential for the role. NUMs reluctant to identify as ‘managers’, instead viewing themselves primarily as nurses. ‐ NUMs devalued and discounted administration and managerial aspects of their work, preferring to discuss their nursing role. ‐ Support structures: All reported lack of support after starting the role. Described difficulty and isolating to be a first‐line manager, relying on peer support group to learn, grow and cope.	100%	Limited transferability, one health authority in one region. Some interviews conducted by telephone.

Pegram et al., (2015), United Kingdom	To explore aspects of working life of ward managers (WMs) and their views regarding their role, perceived challenges and potential enablers to empower WMs to lead clinical care.	Cross‐sectional study. Questionnaire self‐designed, drawing upon published literature.	One large NHS large acute hospital trust in the United Kingdom across 3 sites.	75 WMs.	‐ Low to moderate job satisfaction ‐ Lowest satisfaction related to pay and highest to variety of their work. ‐ Moderate to high occupational stress. ‐ Greatest stress related to time pressure, workload and staff shortages with stress associated with ‘underload’. ‐ Variable organisational commitment to employer; overall organisational commitment was moderately strong ‐ Low response rate to item ‘I am willing to put in a great deal of effort beyond that normally expected in order to help this trust be successful’. ‐ Strongest commitment with not having made a mistake in working for the trust and being willing to put in extra effort beyond that normally expected. Lowest associated with being willing to take any job to stay working within the trust. ‐ WMs agreed with all proposed aspects of the role, confirming multifaceted. Leading by example. ‐ Important aspects of WMs: leadership skills (75.4%), clinical speciality expert (75%), adherence to policies and procedures (70.8%) and autonomous practitioner and decision‐making (64.1%). ‐ 38.5% (*n* = 25) strongly agreed with ‘being a central point of care decisions in the multidisciplinary team’. Maintenance of clinical standards (81.5%). ‐ (82.1% agreed or strongly agreed) that ‘Multiple responsibilities make it hard to keep on top of everything’: Large numbers of clinical audits (62.1%; *n* = 41) and lack of protected time for managerial work (53.0%). 63.7% reported having enough protected time for clinical work. There were varied responses regarding whether were recognised as autonomous practitioners for clinical and managerial decisions. 70% agreed with all potential enablers to being WM, with ways to be supported. ‐ Lowest agreement were apps (computer programs which run on mobile devices such as smart phones and tablets) for clinical training (28.4% strongly agreed; 43.3% agreed) and opportunities to network with other band 7 staff (22.4% strongly agreed; 47.8% agreed).	100%	Low response rate. Self‐reported. Potential response bias.

Rankin et al., (2016), United Kingdom	To explore experiences of senior charge nurses (SCNs) provided with ‘increased supervisory hours’.	Mixed methods. Online questionnaire and semistructured interviews.	Three hospitals in Scotland.	60 SCNs (survey), 12 interviews.	Factors facilitating role of the SCN ‐ Availability and appropriate use of supervisory time: SCNs responded positively to increased supervisory time (22.5 hours/week compared with 7.5 hours/week), enabling them to create ward environments that promoted safety for patients and staff. ‐ Adequate staff capacity: Delegating audit‐related tasks allowed them to focus on priorities while developing team skills and engagement in the audit process. ‐ Preparation and support for the role: Some had well‐developed leadership skills from prior senior roles, while others benefited from workshops, coaching from senior staff and peer networking and support. ‐ Effective leadership skills: Leadership attributes required: motivational skills, time management, conflict management, analytical skills, critical thinking and supportive and approachable personality. Personal traits: desire to improve patient and staff experience, prioritisation, positivity, manage personal stress and pride in the role. SCNs recognised their role model influence, emphasising adherence to workplace rules, clinical expertise, ward visibility and the importance of gaining respect to support staff morale and mentorship. Barriers hindering role of the SCN ‐ Demands and expectations of the role: Representing continuity of care to patients and families. Complex role: ‘juggling’ multiple roles—nurse, educator, ward manager, mentor, role model and supervisor. Role manageable with adequate supervisory time and full staffing. However, increased burden when also managing a patient caseload. ‐ Growing time constraints: Unable to utilise available supervisory time due to increasing complexity of work environment. Worked extra hours, either in the office or at home, to cope with workload and accepted working extra hours was a necessary part of their role. ‐ Managing staffing levels: Managing staffing levels was challenging, with increased time to manage staff rosters and skill mix. Staff absences made rostering difficult, time‐consuming, preventing use of allocated supervisory time to cover rosters. ‐ Support for the role: New SCNs found adjusting to their roles challenging, learning through trial and error, leading to role stress. Lack of clarity about duties, responsibilities and limited opportunities for coaching, mentorship or confidential peer networking to discuss challenges.	86.5%	Study was undertaken following a rapid review involving the hospitals. Increased supervisory time was relatively recent. Findings not generalisable as only reflect on NHS Board in Scotland.

Savage & Scott, (2004), United Kingdom	To understand the experience of matrons in terms of extent of their role and challenges involved in undertaking it.	Multiple methods. Postal survey, semistructured interviews, work diaries.	10 National Health Service trusts in England.	176 matrons (survey), 21 matrons (interviews) and 10 matrons (work diaries).	‐ Matrons with clinical focus, depending on the size of their area, frequently visited clinical sites or provided hands‐on care, engaged in quality improvement by serving as clinical role models, delivering education, and offering supervision. Matrons, less clinically involved influenced care standards by developing nursing guidelines, establishing audit systems and overseeing initiatives like The Essence of Care (Department of Health 2001b). ‐ Most reported spending time on HRM tasks: staff recruitment, managing staff bank and reducing sickness and absence rates. Many felt they were not ‘proper matrons’ due to limited direct patient care or weak connections with patients and families. Three role implementation models, reflecting hybrid management with varying emphasis on matrons’ clinical and corporate responsibilities. ‐ 1st‐Matron was an integral member of the clinical team. Heavily clinical focus, up to 50% of time included in nursing roster, highly accessible to staff, offer model of good practice and well placed to identify nurses’ professional development needs. ‐ 2nd‐Matron had strong clinical focus, supernumerary to nursing team. Clinical work of ad hoc nature, covering during shortages of staff or providing care in response to incidents during matron’s tour of the clinical area. ‐ 3rd‐Matron had little direct clinical involvement besides helping out in times of crisis, largely involved in operational issues or shaping clinical practice through development of standards or protocols.	70%	Findings not transferable.

Scott & Timmons, (2017), United Kingdom	To explore leadership of quality patient care from ward leaders’ (WLs) perspectives and develop an account of WLs experiences of leading.	Qualitative. Semistructured interviews.	One large, acute NHS teaching trust.	19 WLs and modern matron.	3 main themes influenced ward leaders’ leadership experiences: ‘visibility and leading by example’, ‘empty conformity’ and ‘authority and autonomy’. ‐ WLs believed visibility and leading by example were important, reflecting how they managed care quality. ‐ Experienced empty conformity, focussing on corporate goals and targets over patients’ experiences, highlighting a gap between managerial and professional values. They tried to find balance between achieving targets and delivering patient care.	100%	Limited generalisability. Researcher’s role as a trust employee. Using one trust as the study site. Exclusion of other healthcare staff from the sample.

Shirey, (2009), United States	To describe stress and coping as perceived by today’s nurse manager (NM) incumbents.	Qualitative. Structured interviews incorporating components of the critical decision method.	Three acute care hospitals.	21 NMs.	‐ Difficult situations: pressure to perform, interpersonal conflicts related to organisational communication deficits, and human resources and staffing issues. ‐ NMs utilised both emotion‐focused and problem‐focused coping strategies. Novice NMs (3 years or less in role) with experienced NMs (more than 3 years), novices used predominantly emotion‐focused coping strategies, narrow repertoire of self‐care strategies, and experienced negative psychological, physiological, and functional outcomes related to coping efforts. ‐ Experienced NMs working as comanagers demonstrated problem‐focused coping strategies, broad repertoire of self‐care strategies, and reported no negative health outcomes.	100%	Limited generalisability of findings. Cross‐sectional measures only provided a snapshot of the phenomenon of interest. Self‐report data may not have provided a full picture of stress and coping in NMs.

Shirey et al., (2010), United States	To provide a qualitative description of stress and coping as perceived by today’s nurse manager (NM) incumbents.	Qualitative. Individual interview.	3 acute care hospitals in the US (a 528‐bed, magnet‐aspiring facility and 2 facilities not pursuing magnet designation, each with 170 beds).	21 NMs.	‐ Sources of stress Subtheme 1: Situations in general that are sources of stress: People and resources, tasks and work and performance outcomes. 76% identified staffing, financial side (most stressful in first 2 years as NM), and volume of work (wearing many hats, add‐on work and extensive committee work). Subtheme 2: Factors that increase stress: Actual NM work (specific responsibilities of role) and sssues surrounding NM work (peripheral issues arising in role). Not having enough hours in the day, being torn in multiple directions, having excessive committee meetings and experiencing numerous daily interruptions. Subtheme 3: Factors that decrease stress: Focussing on positives, having support from others, ompleting work and achieving targets and incorporating quality downtime. NMs discussed factors that decreased stress, 86% actively pursued ways to decrease stress. Availability of support identified, comanagers reported higher levels of support empowered and assisted in work completion. Subtheme 4: Emotions associated with stress: pure positive emotions, pure negative emotions and mixed emotions. ‐ Coping strategies Subtheme 1: Using a combination of strategies Emotion‐focused vs. problem‐focused coping and narrow vs. extensive repertoires of self‐care strategies. Novice NMs demonstrated used emotion‐focused coping strategies with narrow repertoire of self‐care strategies. Subtheme 2: Experience and differences in coping strategies Coping responses varied based on extent of NM’s experience and employment model: comanager Subtheme 3: Co‐manager model and differences in coping Comanagers used cognitive reframing to put issues into perspective. Comanagers reported strong support and empowerment from their CNO and peers, helping them remain resilient even under extreme stress. ‐ Health‐related cutcomes Subtheme 1: Psychological outcomes NMs reported adverse psychological outcomes: overwhelmed, heightened sense of awareness with frequent exposure to stressful situations. Subtheme 2: Physiological outcomes 86% adverse physiological outcomes resulting from role‐related stress, sleep or staying asleep at home was most common negative physiological outcome reported. Subtheme 3: Functional ability 67% high levels of functioning despite experiencing daily difficult work situations. 33% changes in functional ability related to decreased personal productivity, increased procrastination, and extremes in vigilance (either decreased attentiveness or excessive attention to detail predisposing to exhaustion).	90%	Limited generalisability. Self‐report.

Shirey et al., (2013), United States	To provide a qualitative description of nurse manager (NM) stress and coping experiences.	Qualitative. Individual interviews.	3 acute care hospitals in the US.	21 NMs.	Cognitive decision‐making Subtheme 1: Self‐reflective questions‐NM’s cognitive decision‐making. Subtheme 2: Salient factors that influence cognitive decision‐making (5 identified). These amplified expert/novice differences with cues, pattern recognition, trade‐offs, past experience and data. Subtheme 3: Effects of stress on NM’s cognitive decision‐making (subdivided into 5 categories: decisions at work and home). Inattention to detail due to dealing with multiple simultaneous competing priorities, constantly being challenged to do the impossible and experiencing frequent workflow interruptions; Inability to complete detail‐oriented work presented threat to accuracy and was a personal source of concern. NMs concerned they had made errors that could not be immediately identified but could surface later; as they worked in organisations with lean human resources, they had few capable individuals to whom they could delegate responsibility. Comanagers divided work such that one could always be available to take on additional duties and handle unexpected situations; In addition to work‐based stress issues related to decision‐making, all experienced NMs (76%), but not comanagers reported sleep pattern disturbances; two‐thirds reported high levels of functioning despite experiencing daily difficult situations at work. 34% reported going home after work both physically and mentally exhausted, with no energy left to make decisions.	90%	Limited transferability. Potential response bias.

Smith et al., (2024), South Africa	To explore and describe presence practices amongst unit managers (UMs) in a selected provincial hospital in Free State Province.	Qualitative. Semistructured interviews.	Four provincial hospitals in the Free State.	12 registered nurses (UMs), 10 female, 2 male.	‐ Theme 1: Presence practices among UMs in a selected provincial hospital in Free State. Category 1.1: Leadership presence and accessibility: Importance of being viable, accessible and supportive workplace environments. Category 1.2: Supportive work culture: Ability to create supportive work culture (positive and helpful attitudes recognising diversity) for subordinates and patients by conducting personal interviews before referring them for specific assistance if needed. Category 1.3: Effective management and development: Need for problem‐solving and conflict management skills for smooth operation of unit. They motivated and encouraged subordinates to empower themselves through education and provision of regular in‐service training. UM managers should plan daily schedules actively whilst implementing effective time and resource management to ensure sufficient staff and resources to complete daily unit activities. ‐ Theme 2: Impact of presence practices on hospital dynamics in a selected provincial hospital in Free State Category 2.1: Positive impact on patient care and staff management: Explained how nursing presence improved patient satisfaction and positively affected staff management contributing to team harmony, teamwork, and collaboration. Category 2.2: Challenges and negative outcome: Could lead to patient dissatisfaction and decline in quality nursing care. Lack of UM’s presence could harm the hospital’s image and hinder patients’ and families’ healing. ‐ Theme 3: UMs’ practices of relational care and human connectedness in the unit Category 3.1: UMs’ practices of relational care: Communication integral to practising presence whilst establishing trustworthy and professional relationships and rapport in work environment. Category 3.2: Practices to facilitate human connectedness: Importance of empathy with staff and providing others with necessary support. ‐ Theme 4: Perceptions of UMs on barriers to presence practices in a selected provincial hospital in Free State Category 4.1: Managers and staff‐related barriers to presence practice: Fatigue, burnout, staff attitude and staff members with personal disequilibrium. Category 4.2: Work‐related barriers to presence practices: Insufficient resources, work overload, multiple expectations, staff shortages, inadequate support and lack of acknowledgement. Category 4.3: Barriers to presence practice: Forced to resort to various ways to cope with barriers which could affect practising presence: Relying on others for support, improvisation, self‐sacrifice, emotion‐focused and problem‐focused coping.	100%	Limited transferability‐small sample size.

Surakka, (2008), Finland	To describe and compare characteristics of nurse managers’ (NMs) work in different hospital environments and at different times.	Qualitative. Diaries and focus group interviews.	One health district’s hospital in Finland.	155 NMs.	‐ NMs tackled diverse issues inside and outside the unit and involved organising, cooperating and communicating. ‐ Organising: guiding, supervising nursing staff and care, financial duties, planning, evaluating and organising staff workload assignments and working conditions, patient flow and own work. ‐ Cooperating: staff meetings and cooperation with medical staff, professionals from other clinics, company representatives, hospital safety representatives, shop stewards and quality assurance liaisons. Supporting: assuring skills and competencies of staff and developing performance identified as NMs’ accountability‐related activities: staff’s needs, creating atmosphere, dialogues, reflections and seeing to staff capacity to work. Difficult to delegate and required NMs to always be available for consultation. ‐ Development: evidence and research results, patient satisfaction measurements, staff suggestions for practice improvement, external demands such as organisational, quality improvement work, and management by objectives (MBO) outcomes.	70%	Limited transferability.

Taylor et al., (2015), Australia	To raise nurse managers’ (NMs) critical awareness of practice problems; uncover practice constraints and improve work effectiveness.	Action research. Fortnightly group meetings, for 1 hour, for 10 meetings.	One rural hospital.	3 experienced female NMs.	‐ Phase 1: Identification of ‘being drained by intensity of NMs’ work’. Participants adopted five strategies: debriefing problematic situations; deflecting multiple requests; diffusing issues; naming dysfunctional behaviours; and regrouping. ‐ Phase 2: Implementation and revised action plan strategies, which resulted in them feeling less drained by their work.	90%	Limited transferability, small participant numbers, one rural hospital.

Townsend et al., (2015), Australia	To identify and explore key obstacles preventing ward managers (WMs) from effectively performing human resource management (HRM) responsibilities required in their role.	Qualitative. Interviews.	One private not‐for‐profit hospital transitioning to high performance human resource management (HPHRM) system.	37 (6NMs; 16 nurses; 10: general manager, four directors‐including HR, medical services; support services; and procedural service; 5: middle managers).	‐ Budget pressures: Role and responsibilities of WMs focused on management of budget and employees than in earlier years. 14 key performance indicators (KPIs) of the role reviewed annually. In practice, WM had to balance competing interests: elements of patient care, budget pressures from funding arrangements and hospital expansion led to reduction in the patient/nurse ratio in many wards. Ward‐level patient care changed with greater pressure on ward staff. Budgetary pressures stifled WMs, prevented decision‐making capacity; created conflicting priorities, to meet financial KPIs often came at the expense of effective HRM. ‐ Managerial skills: Formalised training for WMs often left them ill‐prepared for their roles. Most ‘get on’ and deal with managing in their own way. Some engaged in self‐funded managerial and leadership courses and some instigated regular meetings to provide support and share relevant information. Influencing performance on the ward: WMs critical to HRM: building relationships, employee commitment to the ward and WMs, commitment reduced turnover levels and staff efficiencies. Employees held perception that how the WM treated staff impacted on patient care and supporting patients’ families.	90%	Limited transferability, one hospital.

Udod & Care, (2011), Canada	To describe stress experiences and coping strategies of nurse managers (NMs) in an acute care setting as a way to recruit and retain nurses in managerial roles.	Qualitative. Interviews.	Tertiary care hospital in a city in Western Canada.	5 NMs.	Two themes, Identified stressors and coping strategies, with subthemes. ‐ Identified stressors: Financial responsibilities, inadequate human resources, managing others, intrapersonal distress, middle‐management role and competing priorities. ‐ Coping strategies: Peer and supervisor support, cognitive coping strategies, social and personal strategies. NMs felt pressured by senior management to maintain balanced budgets, while union advocated for increased RNs to support less stressful work environment, improve safety and patient care quality. Challenges managing interpersonal staff relationships: suspicion and mistrust of nurses toward management and power struggles. Managers expressed angst, frustration and worry surrounding implementation of their role in varying degrees and intensities: inability to obtain consistent financial and human resources to reduce nurses’ stress, provide safe patient care and ‘letting go’ of high expectations. Felt ‘being sandwiched’, responsible to senior management for implementing organisational policies, meeting goals and organisational priorities, while also responding to staff needs. Nurses stated ‘drowning’ and overwhelmed with work, while supervisors will cite budget deficits to deny additional staffing. NMs struggled with defining boundaries and prioritising work responsibilities: responding to staff and patient needs, organisational priorities, overwhelming amounts of paperwork and email, while trying to maintain clinical presence on the unit. Coping strategies: Vent frustrations, share common experiences, clarify interpretations of organisational changes and share ideas in finding solutions with colleagues. Made conscious choices about when and how to respond to work demands. Balanced work and personal life through coping strategies like exercise, faith, family support and self‐reflection	90%	Limited transferability, small sample size.

Udod & Care, (2012), Canada	To examine stress experiences and coping strategies of nurse managers (NMs) in an acute care setting.	Qualitative. Semistructured interviews.	One large tertiary hospital in Western Canada.	5 NMs.	Identified Stressors Subtheme 1: Fiscal responsibilities: Minimal training to handle financial responsibilities and trouble understanding budgeting process. Subtheme 2: Inadequate Human Resources: Lack of registered nurses to deliver safe, quality patient care. Budgetary concerns influenced shortages as managers tried to adjust schedules while maintaining adherence to nurses’ union collective agreement, limit overtime and manage overworked staff. Subtheme 3: Managing others: Challenges in guiding and supervising interpersonal staff relationships encompassed suspicion and mistrust of nurses towards management and power struggles. Subtheme 4: Intrapersonal distress: Managers’ intrapersonal angst, frustration and worry regarding implementation of their role experienced varying intensities of stressors in their units. Subtheme 5: Middle management role: Described being sandwiched as being caught between staff needs and responsibility to senior management in implementing organisational policies and meeting authorised organisational goals. Coping strategies Subtheme 1: Peer and superior support: Importance of collegial support from first‐line managers and senior management. Immediate supervisors were a source of support and often served as sounding board, but degree of reliance on superiors varied due to superiors having their own demands. Colleagues provided a sounding board to vent frustrations, share common experiences, clarify interpretations of organisational changes and share ideas in finding solutions. Subtheme 2: Cognitive coping strategies: Made conscious choices about when and how to respond to work demands.	100%	One hospital limits transferability. Potential self‐report bias.

Udod et al., (2017a), Canada	To understand nurse managers’ (NMs) perceptions of their role stressors and coping strategies within urban and rural acute care settings.	Qualitative. Individual semistructured interviews and a focus group interview.	Six different acute care facilities in Western Canada representing both rural and urban sites.	17 individual interviews, 5 in one focus group.	Role stressors Subtheme 1: Working with limited resources: Limited financial and human resources in day‐to‐day operation of managing a patient care unit(s) for safe, quality patient care. Subtheme 2: Responding to organisational change: Perceptions of continual flow of priorities and initiatives influencing unit operation that included lean management, enhancing patient flow and improving delivery of care. Subtheme 3: Putting out fires: Responsibility and authority inherent in the role enabled managers to respond quickly to immediate and urgent problems at unit level to ensure safe, quality patient care. Subtheme 4: Senior management’s disconnection: Perceived lack of understanding and support by middle (director) and senior nursing management regarding reality of nurses’ practice environments and extent to which front‐line managers could make change feasible. Subtheme 5: Adhering to regulations and standards: Challenges in following and adhering to organisational regulations and standards associated with quality improvement initiatives and collective agreements. Such policies described as integral, dominating and organising features in a manager’s work. Subtheme 6: Being pulled in different directions: Number and intensity of competing priorities and struggles with psychological demands: organisational, role and relational pressures with superiors, staff and other departments. Inability to focus due to constant mental prioritisation while being frequently interrupted. Coping strategies Subtheme 1: Planful problem‐solving: Proactive approach to problem solving tasks and interactions with staff by being visible, building and supporting relationships to decrease stress and/or prevent issues from arising. Subtheme 2: Reframing situations: Understood coworkers’ pressures and attempted to cope with situations: taking initiative in ‘doing it my way’ and ‘letting it roll off my back’ in developing resilience. Felt as ‘one piece of a very large puzzle’ to share responsibility on a task. Subtheme 3: Having social support from all directions: Importance of psychosocial support and nurturing: from superiors, colleagues, family and friends. All levels of management were significant supports providing sounding boards to vent, share ideas and experiences, provide guidance and advice to navigate intricacies of the role and decompress.	90%	Limited transferability. Potential self‐report bias.

Udod et al., (2017b), Canada	To understand nurse managers’ (NMs) perceptions of their role stressors, coping strategies, and self‐health‐related outcomes as a result of frequent exposure to stressful situations in their role.	Qualitative. Individual interviews and focus groups.	8 care facilities within 2 regions representing both rural sites and urban sites in Western Canada.	23 (individual interviews), 5 (focus groups) NMs.	Role Stressors Subtheme 1: Working With Limited Resources: Working with limited resources (budget and staff) in day‐to‐day operation of managing patient care units. Subtheme 2: Responding to Continuous Change Within Organisational Work Complexities: Cumbersome and inefficient organisational processes, while responding to continual flows of new initiatives including Lean management system, models to enhance patient flow, and improving care delivery. Subtheme 3: Senior Managements Disconnection From Practice. Lack of understanding and support by middle (director) and senior healthcare management regarding reality of practice environments and extent to which NMs could facilitate change and how staff believed things should be done. Coping strategies Subtheme 1: Planful Problem Solving: Assumed proactive approaches to solving problems in tasks and encounters with being visible on the unit, building relationships, and supporting staff. Subtheme 2: Reframing Situations: Psychological process: reflecting, reorienting, and reconciling situations to decrease anxiety and fear. Subtheme 3: Having Social Support: Psychosocial support and nurturing received from superiors, colleagues, family, and friends.	90%	Limited transferability. Potential self‐report bias.

Urban et al., (2023), United States	To describe nurse managers’ (NMs) perceptions of and barriers to professional well‐being during the COVID‐19 pandemic.	Qualitative. Two open‐ended questions—survey.	13‐hospital healthcare system located in a large metropolitan area in the Southwestern U.S.	80 NMs.	Four key themes: (1) Navigating work–life balance: NMs wanted to separate work and home responsibilities without sacrificing or compromising either. Expressed desires to be fully present and effective in their management roles, rather than switching between work and nonwork responsibilities during the day. (2) Investing in yourself: Equated sense of balance, peace or contentment as prerequisite to well‐being. Caring for mind, body, emotional and spiritual self was seen as essential to well‐being, finding it challenging. Investing in balanced self could reduce stressors, improve physical, mental energy and feeling of overwhelmed. (3) Giving and receiving support: Advocate building relationships with and assist in professional development of nursing staff. Wanted to feel supported, trusted and appreciated by direct reports and their own leaders. (4) Thriving versus struggling in the role: Thriving meant feeling good about the work they were doing, managing job demands, experiencing success in the role, having energy to pursue growth or change within the department or feeling passionate, happy, caring or creative in their work. Realistic expectations and maintaining flexibility were key. Three key themes emerged regarding barriers: (1) Time and workload: More time to complete responsibilities: office tasks, time with staff and patient rounding. Multiple action items from various sources created task overload, causing stress, working from home after hours or on weekends to catch up and reducing well‐being. (2) Feeling stuck in the middle: Corporate changes increased as threat from COVID‐19 decreased, creating potentially unrealistic expectations for NMs caught in the middle. (3) Staffing challenges: Changes in nurse employment patterns and staffing challenges exacerbated by COVID‐19. Staff leaving for more pay or different work settings resulted in staffing shortages, while financial challenges from COVID‐19 impacted productivity and staffing ratios on units. Staffing‐related stress, staff dissatisfaction with long work hours or lack of equitable compensation negatively affected well‐being.	90%	Convenience sample from a single hospital system limits transferability. Potential self‐report bias.

Urquhart et al., (2018), Canada	To empirically examine the role of middle managers (MMs) relevant to innovation implementation and how MMs experience the implementation process.	Qualitative. Semistructured interviews.	2 Canadian provinces (Nova Scotia and New Brunswick).	15 MMs who managed hospital‐based programmes, services, departments, or units wherein a cancer‐related innovation was implemented.	3 overarching categories: ‐ Responsible for making implementation happen: Responsible for implementation within their programs and services. Five roles in implementation: planner, coordinator, facilitator, motivator and evaluator. ‐ Other roles and responsibilities: Had clinical alongside managerial duties, faced extra work while fulfilling other roles. Most possessed clinical expertise but lacked formal training in HR, project, or change management, prompting continuous self‐learning. Felt juggling multiple activities hindered optimal performance, a challenge combined by need for additional learning. ‐ Limited decision‐making power: Status quo with limited decision‐making power in decisions to implement innovations, goals and strategies. Senior managers and administrators made decisions, although MMs provided information to assist in these decisions and were expected to work within set parameters. Limited decision‐making authority meant implementations even when they will poorly fit the setting or have minimal impact on patient care.	90%	Limited transferability.

Vasset et al., (2023), Finland, Norway	To emphasise nurses’ experiences of nurse leaders’ changing roles over 25 years.	Qualitative. Individual interviews.	Primary health services or specialist health services in Norway or Finland.	8 nurse managers (NMs).	3 phases of participants’ careers: ‐ Beginning: shared role between clinical work and leadership. In the 1990s, strict hierarchical order in hospitals. Doctors: self‐appointed leader, outlined direction of activities. NMs’: 50% leadership and management and 50% to clinical practice, had to be up to date on clinical practice procedures. First decade: leadership tasks: manageable with opportunity to overview and lead different processes in the workplace. ‐ Middle: leader and manager: Shift in required skills; demanding patients previously cared for in specialist health‐care centres transferred to primary healthcare (PHC), without planned training or supervisor programs implemented for staff. Specialist health service leaders helped PHC leaders manage patient challenges. Staff competence requirements grew, leaders had fewer opportunities for direct contact with nurses as role shifted upward in the hierarchy, focussing more on administration and losing clinical perspective. ‐ Present: nursing leadership: Subordinates listened differently than before. Organisations larger geographical distribution due to merged hospitals, changes simultaneously accelerated, increased virtual meetings, considered both strength and burden. Staff stress‐resistant, creative, open to change, importance of informing and involving them early in change processes.	90%	Limited transferability

Wong, (1998), Hong Kong	To capture work dynamics and impact of nurse managers (NMs) during healthcare restructuring.	Qualitative. Case study. Interviews.	A medium‐sized general hospital in Hong Kong which provided a variety of services.	6 department operations managers (DOMs) and 6 ward managers (WMs).	‐ Patient care: WMs‐12.5%; DOMs‐0%. All WMs had to attend to patient care because of manpower shortage. WMs became involved in clinical work due to responsibility for ward to operate smoothly. ‐ Student supervision: WMs was 1.7%, DOMs‐ 0%. Day‐to‐day operational management: WMs‐51.6%; DOMs‐38.0%. Communication: 12% WMs and DOM. Planning, quality improvement and budgets: WMs‐10.0%; DOMs‐41.0%.	80%	One hospital limits generalisability.

The most frequently utilised data collection method was semistructured interview (*n* = 30), followed by questionnaire (*n* = 8), direct observation (*n* = 2), focus group (*n* = 1) and coaching session (*n* = 1). Several studies used combined methods, including group meeting with journal reflection (*n* = 1), semistructured interview with focus group (*n* = 1), semistructured interview with direct observation (*n* = 1), focus group with questionnaire (*n* = 1), interview with questionnaire (*n* = 1), semistructured interview with direct observation and dialogue forum (*n* = 1) and diary methods with semistructured interview (*n* = 1).

NMs were referred to by 18 different titles, including unit manager, ward manager, NM, first‐line NM, middle manager, matron, senior charge nurse, ward sister, charge nurse, midlevel NM, clinical first‐line NM, organisational middle manager, head nurses, ward leader, modern matron, nurse unit manager, nurse and midwifery unit manager and nursing unit manager; however, the essence of their roles remained similar. Sample sizes in the included studies ranged from 3 to 433, some intentionally conducted at microlevel to capture experiences of how NMs balance clinical and managerial responsibilities, while quantitative studies were conducted with larger sample sizes. This review represents 1680 NMs across included studies; however, one study did not clearly specify participant numbers [35].

### 4.2. Findings

Thematic analysis identified three main themes and five subthemes: (1) *key challenges faced by NMs in fulfilling both clinical and managerial responsibilities*, comprising navigating complex role demands and organisational and workforce constraints; (2) *strategies used by NMs to balance dual roles,* including emotion‐focused strategies and cognitive‐focused strategies; and (3) *factors influencing NMs’ prioritisation and decision-making,* consisting of *clinical and organisational influences on prioritisation*. Table 4 outlines the themes, subthemes and illustrative examples.

**TABLE 4:** Themes, subthemes and illustrative examples.

Themes	Sub themes	Illustrative examples	
Key challenges faced by nurse managers in fulfilling both clinical and managerial responsibilities	Navigating complex role demands	• Varied roles and responsibilities • High workload, stress and dissatisfaction • Expanding roles expectations • Competing priorities • Role ambiguity and role confusion • Organisational complexity and change	• Middle‐management role challenges • Managing others • Managing high‐performance expectations • Financial responsibilities • Dual‐role complexities
	Organisational and workforce constraints	• Ineffective communication • Lack of role preparedness • Inadequate human resources • Regulatory and organisational compliance demands • Information system challenges • Leadership issues	• High responsibility with limited authority • Perceived lack of value • Insufficient support and understanding • Job dissatisfaction • Intrapersonal distress • Personal sacrifices

Strategies used by nurse managers to balance dual roles	Emotion‐focused strategies	• Influence of professional experience • Assistance and support • Reframing situations	• Past professional experiences • Work–life balance
	Cognitive‐focused strategies	• Planful problem solving • Deliberate decision‐making • Role distancing	• Leadership presence and accessibility • Leadership skills

Factors influencing nurse managers’ prioritisation and decision‐making	Clinical and organisational influences on prioritisation	• Clinical presence • Supporting staff • Patient and organisational needs • Staff shortages	• Models of care • Role responsibility • Professional identity • Unit work model

#### 4.2.1. Theme 1: Key challenges Faced by NMs in Fulfilling Both Clinical and Managerial Responsibilities

Increasing demands and growing complexity of healthcare systems have positioned NMs at the forefront of care delivery, given their critical influence on healthcare outcomes, patient safety, and organisational effectiveness [2, 11]. Forty‐six (94%) studies identified key challenges faced by NMs in fulfilling both clinical and managerial responsibilities. Two subthemes emerged within this theme. The first, *navigating complex role demands*, reflects the multifaceted and evolving nature of the role. The second subtheme, *organisational and workforce constraints,* captures the structural and systemic factors that limit NMs’ capacity to effectively balance clinical and managerial responsibilities.

##### 4.2.1.1. Subtheme 1: Navigating Complex Role Demands

Navigating complex role demands highlights the complexity and breadth of the NM role, characterised by multiple, often competing responsibilities across clinical, managerial and leadership domains, alongside increasing organisational expectations. These findings illustrate the dynamic nature of the role, requiring NMs to continually adapt to diverse responsibilities and competing priorities across practice settings.

This subtheme encompasses a range of interrelated challenges, including varied roles and responsibilities, high workload, stress and dissatisfaction, expanding role expectations, competing priorities, role ambiguity and role confusion, organisational complexity and change, middle management role challenges, managing others and high‐performance expectations, financial responsibilities and dual‐role complexities.

###### 4.2.1.1.1. Varied Roles and Responsibilities

Sixteen (33%) studies highlighted the wide range of responsibilities NMs navigate daily. These included administrative and human resource tasks such as staff recruitment and selection, roster and payroll management, leave approvals and monitoring sickness and absence, alongside financial responsibilities such as budget planning, procurement, invoicing and cost calculation [36–38]. El Haddad et al. [1] reported that NMs spent an average of 25 h per week on administrative tasks, equivalent to 0.6 of a full‐time role, with payroll, rostering, reporting, clerical work, recruitment, mandatory training and stock management being the most time‐consuming activities [1].

NMs were also actively engaged in intradisciplinary and interdisciplinary collaboration within their organisations and the broader community. They undertook advocacy and liaison roles to support patient care improvements, contributed to quality improvement initiatives and facilitated project work [36, 38–41]**.** In addition, they influenced care standards through multidisciplinary decision‐making, participated in developing nursing practice guidelines, challenged poor practices, led audits and managed care delivery [37, 38, 40–42]. Some worked within complex matrix systems, holding responsibility across medical and surgical programme streams while also acting as site supervisors across multiple hospitals [40].

Clinical and educational responsibilities included functioning as clinical experts, delivering education and providing supervision to students, newly qualified staff and multidisciplinary teams across inpatient settings with varied skill mixes [38, 41, 43–46]. Despite extensive managerial duties, many NMs continued to undertake clinical shifts, identifying direct patient care as a core responsibility to support both patients and staff and address immediate concerns. They were frequently regarded as key sources of guidance for clinical tasks and decision‐making [38, 40, 46]. However, managing patient caseloads alongside managerial demands contributed to significant workload pressures, making it difficult to sustain optimal performance across all aspects of the role [36–48].

NMs described their position as a critical link between frontline services and the broader organisation, facilitating communication and interpreting organisational changes for staff [37]. Their leadership responsibilities included coordinating workflows, engaging stakeholders, fostering collaboration, supporting team performance and promoting staff development [49, 50]. Additional expectations included demonstrating accountability, exercising autonomous decision‐making, adhering to policies and procedures and maintaining visibility through formal and informal rounding to understand staff experiences [38, 41, 46].

Over time, managerial expectations expanded to include greater responsibility for patient care oversight, staff leadership, administration and resource management [47]. The role is widely perceived as highly complex, requiring NMs to balance multiple functions as clinicians, educators, leaders, mentors and coordinators [41, 43, 44]. These combined demands increase workload and time pressures, particularly as NMs strive to maintain clinical competence while fulfilling leadership responsibilities, often limiting their ability to effectively meet all aspects of the role [50, 51].

###### 4.2.1.1.2. High Workload, Stress and Dissatisfaction

Twelve (25%) studies identified high workloads, stress and dissatisfaction as key challenges faced by NMs in fulfilling both clinical and managerial responsibilities. A study of rural NMs [39] highlighted distinct challenges compared with metropolitan counterparts, with individuals often managing a broad range of duties independently, including oversight of entire facilities. Increasing remoteness was associated with fewer managerial positions, resulting in heavier workloads, limited human resource support and greater professional isolation. These conditions were compounded by insufficient managerial training, limited support systems and the pressures of an ageing workforce, further discouraging younger nurses from pursuing these roles [39].

NMs reported difficulty effectively utilising supervisory time due to competing demands, including increasing patient complexity, higher caseloads, growing numbers of frail and elderly patients, heightened expectations from patients and families, high bed occupancy, larger multidisciplinary teams and concurrent ward rounds [39, 44]. Additional pressures included high volumes of work, extensive committee involvement and the expectation to assume multiple roles simultaneously [46, 52]. Many also undertook patient assessments during routine shifts and were frequently required to provide unplanned clinical care alongside managerial duties [53]. Nonclinical responsibilities, particularly administrative and human resource tasks, were described as burdensome, with NMs often expected to assume tasks beyond their formal role or those avoided by others [46]. These cumulative demands contributed to workload imbalance and time constraints, limiting opportunities for clinical supervision, staff engagement and patient rounding [40, 43, 50, 53–55].

Increasing complexity of the role contributed to significant physical and psychological strain, including exhaustion, burnout, job dissatisfaction and concerns regarding staff retention [46]. NMs with broader spans of control described leaving work physically and mentally depleted, with diminished capacity for further decision‐making [56]. Stressors arose from core role responsibilities and environmental pressures within the workplace [52]. These demands resulted in psychological (e.g., feeling overwhelmed and hypervigilant), physiological (e.g., sleep disturbances) and functional effects (e.g., reduced productivity, procrastination and fluctuating attention), often culminating in persistent fatigue [52, 56]. Despite these challenges, many NMs maintained high levels of functioning [56].

With experience, NMs developed adaptive skills in task organisation, time management, communication and decision‐making, particularly within their designated service areas or departments in relation to policy and workflow adjustments [57]. Although some reported ongoing feelings of inadequacy, they used strategies to respond to staff needs [46] and engaged in peer support networks to share knowledge and solutions [53].

###### 4.2.1.1.3. Expanding Roles Expectations

Six (12%) studies identified challenges associated with expanding NMs roles, as responsibilities shifted from primarily clinical to predominantly managerial in response to directives from operational management and broader medical, socioeconomic and political changes [39, 42, 47]. It is important to distinguish between role extension and role expansion within nursing practice. Role extension refers to addition of tasks and increased complexity within an existing scope of practice, whereas role expansion involves new areas of responsibility accompanied by greater professional autonomy and decision‐making authority [58–61]. Consistent with these concepts, NMs experienced significant growth in their roles over time. Existing responsibilities including staff leadership, administration and resource management became increasingly complex, while new expectations involved greater responsibility for measuring patient outcomes, managing financial constraints and navigating evolving technologies and organisational change [42, 51, 62]. Decentralisation of authority further intensified these demands, requiring greater managerial accountability and increased engagement with senior leaders and peers [42, 47]. As roles evolved, required skill sets also shifted, with NMs moving further up the organisational hierarchy, reducing direct clinical engagement and focussing more heavily on administrative responsibilities [51].

Workforce shortages, limited training and supervision and increasing demand for skilled staff often required NMs to step back into direct patient care, particularly for complex cases transferred from specialist services. This contributed to increased workload, extended working hours and accumulated stress, as core responsibilities remained incomplete [46, 51]. Rural NMs frequently described themselves as ‘jack‐of‐all‐trades’, undertaking broader and more unpredictable duties than their urban counterparts due to service gaps, professional isolation and varying levels of rurality [39]. Managing patient caseloads alongside expanding responsibilities further intensified role strain, making it difficult to maintain effectiveness across all aspects of the role [42].

Despite these challenges, NMs emphasised professionalism as central to maintaining nursing standards and adapting to organisational demands. Management was viewed as an opportunity for professional growth, particularly through supervising and guiding staff. To reinforce their nursing identity, NMs prioritised visibility, engaged in formal and informal staff interactions and fostered supportive workplace relationships [42].

###### 4.2.1.1.4. Competing Priorities

Eleven (23%) studies identified competing priorities as a key challenge for NMs in balancing clinical and managerial responsibilities. They faced numerous and often conflicting demands [41, 47, 63, 64], including meeting organisational expectations such as implementing directives from multiple sources to achieve performance goals while simultaneously supporting overwhelmed staff [54, 63, 64]. Ongoing organisational changes further intensified these pressures, creating increasingly unrealistic expectations [54, 55]. Many described feeling ‘pulled’ in different directions, positioned between frontline staff and operational management [54, 64]. These competing demands contributed to heightened psychological strain, increased workload and limited time to complete essential tasks, including mentoring, assessing and supporting newly qualified staff [40, 46, 53, 54, 64, 65]. NMs frequently experienced continuous interruptions and ‘mental juggling’, making it difficult to concentrate on specific tasks [56, 64] and maintain a consistent presence in practice [55]. Collectively, these pressures negatively affected their well‐being [40, 46, 53, 54, 64, 65].

###### 4.2.1.1.5. Role Ambiguity and Role Confusion

Nine (18%) studies reported role ambiguity among NMs, particularly in differentiating leadership from managerial, clinical and resource management functions. Limited role clarity, including unclear expectations, requirements and guidance, contributed to confusion, with some NMs interpreting routine managerial activities as leadership [37, 49, 66].

The scope of the role was described as extensive and poorly defined boundaries, combined with limited coaching, further exacerbated uncertainty. As a result, many assumed responsibilities beyond their scope and paid hours, often feeling they had become ‘everything to everyone’ within their service areas [44, 45]. Role transitions were frequently experienced as challenging, with many relying on trial‐and‐error learning, contributing to ongoing role‐related stress [44]. Some also perceived their clinical expertise as underutilised in managerial positions [53].

Several NMs expressed a preference for identifying as nurses rather than managers, often prioritising direct patient care and operational tasks over leadership, administrative and managerial functions [63–68]. Nursing was viewed primarily as a caring, patient‐centred profession, in contrast to the financial and performance‐driven focus of management. This distinction reinforced their commitment to patient care while highlighting tension in adopting managerial identities that emphasise efficiency over empathy [35].

###### 4.2.1.1.6. Organisational Complexity and Change

Five (10%) studies highlighted challenges NMs faced in managing organisational change and complexity. Organisational expectations emphasised responsibilities and accountability in executing directives from operational management [12, 42]. NMs described navigating complex organisational processes, including structural barriers, institutional limitations, financial constraints and inadequate physical infrastructure, while simultaneously responding to continuous organisational initiatives such as lean management systems, patient flow models and care delivery reforms [12, 43].

During COVID‐19, NMs reported shifts in their roles, increasingly undertaking clinical duties and functioning more as frontline nurses rather than in managerial capacities [69]. They also expressed frustration with the rapid pace of change, as evolving provider models and departmental restructuring contributed to role confusion and a loss of control. Ongoing disruption left many feeling unprepared to respond effectively to these changing demands [38, 69]. In addition to these pressures, NMs encountered workplace conflict, including disputes with union representatives, and reported frustration with support services, particularly in relation to recruitment delays [43].

###### 4.2.1.1.7. Middle‐Management Role Challenges

Eight (16%) studies highlighted significant pressures associated with the middle‐management role. NMs are conceptualised as middle managers, positioned between executive leadership and frontline clinicians, fulfilling both administrative and clinical leadership functions. They play a key role in translating organisational strategy into practice while also relaying frontline insights to organisational decision‐makers [65, 67].

One study examined their engagement using the Advanced Practice Role Delineation (APRD) tool, a validated self‐assessment survey measuring NMs’ levels of engagement across five domains of practice. Clinical NMs in hybrid roles reported high engagement across all domains, especially in direct clinical care activities, whereas organisational NMs demonstrated lower clinical involvement, reflecting a shift toward strategic responsibilities. Although both groups contributed to facilitating care, clinical NMs scored higher in systems support domain, as measured by the APRD tool, reflecting their dual role in strategic and direct clinical care activities [70].

In emergency settings, command‐and‐control structures and limited autonomy further constrained NMs, making it difficult to balance clinical oversight with operational demands in high‐pressure, efficiency‐driven environments [38, 65, 71]. NMs frequently described feeling isolated and ‘sandwiched’ between the needs of staff and organisational expectations, while acting as key links across different levels of the organisation [37, 65, 70, 72]. These pressures were compounded by competing demands, time constraints and frequent interruptions, creating a persistent sense of being pulled in multiple directions. Such challenges intensified following COVID‐19, as organisational changes introduced increasing, and at times unrealistic, expectations [52, 54].

###### 4.2.1.1.8. Managing Others

Nine (18%) studies discussed challenges NMs faced in managing others. A key managerial function in maintaining effective practice environments involved managing difficult staff [47]. Changes in staff expectations required NMs to continually adapt their leadership approaches [51]. They often encountered hostility, frustration and emotional pressure from staff, particularly during periods of organisational and workforce strain, and were frequently the focal point for concerns raised by staff, physicians and families regarding care quality [39].

Staffing‐related tensions, including mistrust of management, power struggles, dissatisfaction with long work hours and perceived inequities in compensation, further impacted NMs’ well‐being. Interpersonal challenges, such as negative staff attitudes and personality clashes, disrupted team cohesion and made it difficult to maintain supportive engagement and effectively resolve issues [40, 55]. NMs were often required to mediate conflicts within their teams [40]. Collectively, these challenges contributed to feelings of strain and lack of support [39, 54, 65, 73], further weakening relationships with staff and multidisciplinary teams [11].

###### 4.2.1.1.9. Managing High‐Performance Expectations

Challenges related to managing high expectations emerged in three (6%) studies. Although NMs demonstrated a clear understanding of their roles, they reported feeling overwhelmed by organisational pressures and expectations of staff, colleagues, patients, families and supervisors including directors of nursing [74–76]. They described feeling emotionally exhausted by unrealistic and impractical expectations, requiring them to manage multiple competing demands within limited timeframes, making it challenging to meet all requirements effectively [74–76]. NMs were frequently burdened by concerns raised by staff, physicians and families regarding quality of care, alongside expectations to increase their involvement in direct patient care activities while simultaneously responding to escalating organisational demands [76].

###### 4.2.1.1.10. Financial Responsibilities

Financial responsibilities were identified as a major source of distress for NMs in nine (18%) studies, largely due to the inability to secure consistent financial and human resources needed to reduce staff stress and maintain safe patient care. These constraints limited their capacity to effectively fulfil clinical and managerial responsibilities [37, 65].

NMs experienced competing pressures from operational management and organisational performance expectations to maintain balanced budgets, alongside union demands for increased RN staffing levels to support staff well‐being and ensure safe, high‐quality care [63–65]. These challenges were compounded by expectations to meet new targets despite ongoing financial constraints and limited control over budgets managed by nonclinical personnel, restricting their ability to respond to frontline needs [37, 38, 40, 63]. The COVID‐19 pandemic further intensified these pressures by negatively affecting productivity and staffing ratios [54].

NMs identified staffing and financial constraints as key stressors, particularly during the first 2 years of tenure, spanning domains of people and resources, workload and performance outcomes [52]. In addition, NMs were expected to deliver emergency care for critically ill patients while ensuring cost efficiency and productivity, further exacerbating role demands [71].

###### 4.2.1.1.11. Dual‐Role Complexities

Ten (20%) studies reported that NMs’ roles were significantly more diverse than outlined in formal job descriptions, requiring integration of both clinical and managerial functions [38, 45, 71]. While clinical responsibilities were often prioritised, particularly when working directly with staff to ensure high‐quality patient care and uphold patient‐centred values [47], many struggled to reconcile tensions between clinical and managerial demands. Dual‐role responsibilities were frequently described as overwhelming, conflicting and difficult to integrate, requiring continual adaptation to differing clinical, managerial and leadership expectations [35, 75, 77–79].

Despite these challenges, NMs played a critical leadership role in managing service delivery, acting as agents of change, providing mentorship and feedback, guiding teams through challenges, fostering cohesion and supporting effective decision‐making. However, balancing responsibilities as advocates for patients and staff remained difficult, particularly in the context of limited resources and information, which further intensified role strain [38, 45, 77]. Maintaining clinical presence was often a deliberate strategy to preserve professional identity, with many prioritising patient contact over administrative and leadership tasks [35, 77]. Administrative and leadership demands were perceived to limit NMs’ capacity to function as clinical role models and mentors [78], resulting in feelings of incomplete role fulfilment due to reduced time for patient care and diminished engagement with patients and families [38, 77]. During the COVID‐19 pandemic, many NMs experienced role shifts towards frontline clinical duties. Key challenges during this period included infection control, maintaining communication with families and managing disrupted patient–family interactions [11].

##### 4.2.1.2. Subtheme 2: Organisational and Workforce Constraints

Organisational and workforce constraints reflect systemic and structural conditions that shape how NMs fulfil their role. These findings highlight how limitations in organisational systems, workforce capacity and support mechanisms contribute to role strain and influence performance. This subtheme includes challenges such as ineffective communication, lack of role preparedness, inadequate human resources, regulatory and organisational compliance demands, information system challenges, leadership issues, high responsibility with limited authority, perceived lack of value, insufficient support and understanding, job dissatisfaction, intrapersonal distress and personal sacrifices.

###### 4.2.1.2.1. Ineffective Communication

Communication challenges were identified in three (6%) studies. Ineffective interprofessional communication contributed to misunderstandings regarding staff capabilities, disrupted patient transfers and highlighted organisational communication gaps that affected healthcare delivery [47, 49]. During the COVID‐19 pandemic, these issues were further exacerbated, as communication with teams, patients and families relied heavily on digital platforms and phone‐based interactions, limiting personal engagement and complicating staff management. These challenges reduced communication effectiveness and further constrained NMs’ ability to coordinate care and support staff [11].

###### 4.2.1.2.2. Lack of Role Preparedness

Ten (20%) studies identified that NMs’ roles were considerably broader than outlined in formal job descriptions, requiring competencies distinct from clinical expertise. While NMs were skilled in patient and family care, many reported limited preparation in human resource management, financial and budget management, project management and change management, which are essential for leading staff and implementing organisational initiatives [40, 47, 48, 53, 57, 70, 75, 77].

Transitioning into managerial role was described as challenging, with formal training often insufficient to meet the full scope of role demands. As a result, many relied on experiential learning and ongoing professional development to build the capabilities required for effective management [47, 57, 63, 67]. Many also lacked formal managerial qualifications, despite these being considered essential or strongly encouraged within the role [63].

Collectively NMs expressed feeling overwhelmed, underprepared and unsupported during role transition and throughout their tenure [40, 67]. At the same time, many found it difficult to relinquish clinical responsibilities, reflecting ongoing tension between managerial expectations and professional identity [77].

###### 4.2.1.2.3. Inadequate Human Resources

Ten (20%) studies highlighted challenges related to inadequate human resources, including staffing and budget constraints, which limited NMs’ capacity to effectively fulfil their roles, particularly in workforce planning and maintaining appropriate skill mix [44, 47, 64, 80]. Staff absences due to illness further compounded these pressures, increasing time and effort required for workforce management. As a result, NMs often struggled to utilise supervisory time and maintain consistent presence in their service areas, frequently prioritising roster coordination or stepping in to provide direct patient care [44, 55].

Persistent shortages of RNs remained a significant concern, with budget limitations exacerbating workforce instability. NMs were required to balance staffing schedules, comply with industrial relations agreements and workforce regulations, limit overtime and support increasingly overworked staff [73]. These challenges were further characterised by insufficient equipment, staffing disparities across hospital sites, inadequate multidisciplinary and support staff and limited access to financial information [40].

NMs also reported frustration with on‐call responsibilities and the need to respond to ongoing staffing constraints, highlighting tensions between organisational expectations and frontline clinical demands. One manager described the on‐call role as the ‘most uncomfortable part of the job because you can’t make resources appear’ [65]. These pressures escalated during the COVID‐19 pandemic, as staff attrition, driven by better pay or alternative work settings, further reduced workforce capacity [54]. Concurrently, budget constraints and reported negative variances limited opportunities to recruit additional staff, while shortages of experienced personnel restricted effective delegation, particularly in resource‐constrained organisations [65]. In response, some NMs adopted adaptive approaches, such as task‐sharing between comanagers to maintain coverage for unexpected demands [56].

###### 4.2.1.2.4. Regulatory and Organisational Compliance Demands

Six (12%) studies identified challenges associated with adhering to organisational regulations, standards, quality improvement initiatives and collective agreements, which were described as central components of the managerial role [64]. NMs often described their roles as ‘not easy’, highlighting challenges in balancing patient care and staff leadership, particularly in relation to adherence to organisational policies, procedures, standards and guidelines, and navigating strong union influence on leadership practice [11, 47, 78].

During the COVID‐19 pandemic, work policies were often poorly adapted, hindering service development and project implementation and affecting nurses’ professional functioning in both positive and negative ways [11]. Bureaucratic constraints further limited NMs’ leadership capacity, with some reporting difficulty supporting organisational decisions they felt uncertain about while still being expected to present them positively to their teams. These compliance demands further constrained NMs’ autonomy and contributed to role strain within increasingly complex organisational environments [49, 81].

###### 4.2.1.2.5. Information System Challenges

Two (4%) studies reported challenges related to time‐consuming electronic systems, including unlinked databases, reliance on manual processes due to limited digital infrastructure, inadequate training in system use, insufficient IT support and pressure to implement technological changes mandated by senior management. These system‐related constraints reduced efficiency and increased administrative burden, further limiting NMs’ capacity to effectively coordinate services and support staff [40, 42].

###### 4.2.1.2.6. Leadership Issues

Eleven (22%) studies highlighted challenges associated with the leadership dimensions of the NM role. These included managing emotionally demanding responsibilities, responding rapidly to service‐level issues and maintaining accountability for safe, high‐quality care. Ongoing organisational initiatives, such as lean management, patient flow improvements and care delivery reforms, continually reshaped unit operations [51, 62, 64]. Effective leadership required visibility, accessibility and role modelling to influence staff behaviour and sustain a safe work environment [47]. However, increasing system complexity, including hospital mergers and broader geographic service structures, made it more difficult to engage staff early in change processes [51].

One study reported that NMs demonstrated strong leadership capabilities in engaging others, driving innovation and achieving outcomes; however, they showed lower proficiency in self‐leadership and system‐level influence. Although highly dedicated and absorbed in their work, they reported lower levels of vigour. Engagement was slightly higher among those with postregistration qualifications, while perceptions of organisational support were moderate overall and more positive among newly appointed managers than those with longer tenure [1].

Ward staff emphasised the vital role of NMs in fostering relationships, supporting staff commitment and positively influencing retention, operational efficiency, patient care and family support [63]. However, increasing managerial responsibilities and mandatory meetings often reduced NMs’ presence within their service areas, limiting opportunities for engagement and social support [42, 51]. Reduced visibility and engagement were associated with negative outcomes, including patient dissatisfaction, reduced care quality, delayed recovery and potential loss of public trust in nursing and organisational reputation [49, 55, 69]. Additional challenges influencing leadership behaviours included unconscious anxiety, power differentials, inconsistent collaboration with physicians, limited early professional socialisation and ongoing clinical practice reform [51, 78].

###### 4.2.1.2.7. High Responsibility With Limited Authority

Four (8%) studies highlighted increased responsibility alongside limited authority. NMs were actively involved in multidisciplinary coordination with medical, allied health, pharmacy and support staff, contributing significantly to patient care and service functioning; however, they often lacked formal authority over these groups [40].

Despite holding extensive responsibilities, NMs reported limited decision‐making power regarding innovation, implementation goals and strategic direction and restricted control over key resources such as budgets, for which they remained accountable [53, 77]. They were frequently required to provide input to support operational management decision‐making while working within predefined parameters, often implementing changes they perceived as ineffective or misaligned with patient care. This, in turn, reduced their support for such initiatives [48].

###### 4.2.1.2.8. Perceived Lack of Value

NMs reported feeling undervalued, excluded and isolated, often positioned between organisational expectations and staff concerns [37, 62]. Six (12%) studies highlighted mismatches between high role demands and inadequate compensation, discouraging uptake of these positions, as reflected in the sentiment: ‘step up in responsibility and step down in pay’. Although NMs were recognised as experts and integral to organisational culture, change initiatives, quality care and staff support, this was not reflected in remuneration, support during absences or access to professional development opportunities. Consequently, many perceived the role as lacking appropriate recognition for the personal sacrifices it required [43, 45, 53, 82].

###### 4.2.1.2.9. Insufficient Support and Understanding

Lack of support was a recurring issue, identified in 12 (25%) studies. NMs reported unmet expectations and limited understanding and validation from superiors after assuming the role, frequently describing the position as difficult and isolating at the first‐line manager level [36, 43, 53, 67, 74]. Many expressed a strong need for formal mentorship, noting its absence for both newly appointed and experienced managers. NMs viewed assigned mentors as valuable for guidance in navigating practice realities, managing staff expectations and assessing the feasibility of implementing change [12, 64]. However, time constraints often limited opportunities for mentorship from experienced managers [44, 81].

Insufficient support extended across multiple areas, including emotional reassurance during periods of high workload, staffing and administrative assistance, guidance in managing difficult staff and performance issues, access to resources for implementing change, involvement in decision‐making and support from other departments during role preparation [36, 43, 47, 55]. Concerns were raised about NMs’ time management when expectations went unmet, despite excessive demands and the challenges of balancing clinical, administrative and financial responsibilities, compounded by the absence of adequate support from superiors [43, 75].

###### 4.2.1.2.10. Job Dissatisfaction

Three (6%) studies reported job dissatisfaction among NMs, with participants describing low to moderate levels of job satisfaction. Dissatisfaction was primarily associated with pay rates, while higher satisfaction was linked to the variety of tasks within the role [41]. NMs also reported limited opportunities for ongoing personal and professional development [53], and tensions between managerial duties and clinical engagement contributed to feelings of disconnection from core nursing practice, resulting in role strain and dissatisfaction [76].

###### 4.2.1.2.11. Intrapersonal Distress

Fourteen (29%) studies reported that NMs experienced varying degrees of stress, often characterised by intrapersonal distress, frustration and worry as they attempted to meet role demands [11, 65, 66, 73]. Although they had clear understandings of their role, they frequently felt overwhelmed by organisational expectations and the need to manage underlying systemic pressures [36, 38, 57, 63, 74]. Many reported fatigue, burnt out and frustration stemming from their inability to address staff concerns or resolve ongoing issues effectively, alongside increasing task demands, expanding responsibilities and limited capacity to maintain a visible presence [11, 49, 55, 66]. Constant workflow disruptions further contributed to feelings of unpreparedness and concern about the emotional impact on staff, patients and families. As a result, some experienced a loss of credibility and struggled to rebuild trust amid persistent negativity, making it difficult to remain supportive and motivated [36].

Additionally, many NMs found managing their own high expectations contributed to increased intrapersonal strain, frustration and difficulties adapting to the growing complexity of the role [65, 80]. Some expressed concerns about their ability to complete work to the desired standard, noting that errors were not always identified promptly and could compromise accuracy over time [56]. NMs also reported that previously effective strategies had lost their impact, leaving them struggling to lead disrupted teams, provide emotional support and make well‐aligned decisions with operational management expectations [38, 63]. Reduced time in their service areas due to increasing numbers of meetings and off‐site discussions further distanced them from direct clinical engagement [37]. Many described feelings of loneliness, emotional strain, low mood and challenges in maintaining work–life balance, with several becoming emotional when reflecting on their experiences. Experienced NMs, in particular, reported declining motivation and a lack of clear career direction [11, 36, 57, 66].

###### 4.2.1.2.12. Personal Sacrifices

Five (10%) studies reported that NMs made personal sacrifices to balance clinical and management responsibilities. Meaningful work was identified as a key factor influencing both attraction to and retention in the role, supported by a sense of passion, pride and opportunities for ongoing professional growth [43].

Personal sacrifices included extended working hours and reduced time with family to meet professional demands, maintain service area functioning and achieve positive outcomes [47, 82]. Many worked beyond contracted hours, both on‐site and at home, often viewing this as an integral expectation of the role [44, 47, 53]. Some also used personal funds to purchase supplies to support service area operations. Gender differences were noted, with male managers forgoing additional income‐generating opportunities, while female managers arranged additional childcare to accommodate increased work commitments [82].

#### 4.2.2. Theme 2: Strategies used by NMs to Balance Dual Roles

Complexity of NMs’ roles arises from the need to balance clinical and managerial responsibilities simultaneously. The demands associated with both roles often result in role ambiguity, conflict and overload, contributing to stress and dissatisfaction among NMs [8, 14]. In response, NMs employ a range of strategies to manage these competing demands and maintain role effectiveness.

Twenty‐four (49%) studies explored strategies used by NMs to balance their dual roles. These strategies reflect adaptive responses to the challenges encountered in practice, encompassing both emotional coping mechanisms and structured, problem‐oriented approaches. Two subthemes emerged within this theme. The first, *emotion-focused coping strategies,* highlights approaches NMs use to manage emotional demands of the role. The second subtheme, *cognitive-focused coping strategies,* reflects practical and decision‐making approaches used to manage responsibilities and competing priorities.

##### 4.2.2.1. Subtheme 1: Emotion‐Focused Strategies

Emotion‐focused coping strategies highlight the emotional demands associated with balancing clinical and managerial responsibilities. These strategies reflect efforts to regulate stress, maintain resilience and adapt to ongoing psychological pressures within complex and resource‐constrained work environments. This subtheme includes strategies such as influence of professional experience, assistance and support, reframing situations, past experiences and maintaining work–life balance.

###### 4.2.2.1.1. Influence of Professional Experience

Two (4%) studies reported that coping strategies varied according to NMs’ level of professional experience. Newly appointed NMs (3 years or less in role) predominantly used emotion‐focused coping strategies with limited repertoire of self‐care strategies and experienced negative psychological, physiological and functional outcomes. In contrast, more experienced NMs were more likely to use problem‐focused coping strategies, drawing on a broader range of self‐care approaches and reporting fewer negative health outcomes [51, 80].

###### 4.2.2.1.2. Assistance and Support

Seeking assistance and support to balance dual roles was identified in 13 (27%) studies. NMs relied on collegial, psychosocial and nurturing support from family, friends, colleagues and all levels of management. These support provided opportunities to express frustrations, share ideas and experiences, clarify interpretations of organisational changes, receive guidance in navigating role complexities and alleviate stress [12, 64, 65, 73]. Coaching, regular meetings, peer networking and group support were crucial in facilitating learning, professional growth and ability to cope with job demands [44, 45, 63, 77].

NMs also accessed broader resources, including organisational systems, religious practices and spiritual support to manage challenges and build resilience [40, 78]. The support received enhanced efficiency at individual and organisational levels, promoting job satisfaction, fostering collaborative problem‐solving, supporting work–life balance and facilitating ongoing professional development [46]. Availability of support was considered crucial, as NMs emphasised the importance of feeling trusted, valued and supported by direct reports and senior leaders [54], enabling them to manage workload demands, maintain composure in high‐pressure situations and contribute to organisational goals [52].

###### 4.2.2.1.3. Reframing Situations

Nine (18%) studies described how NMs used cognitive reframing, a psychological process involving reflection, reorientation, improvisation, adaption and reconciliation of situations to gain perspective and reduce anxiety and fear [12, 52, 56, 64]. This aligns with self‐leadership theory, particularly cognitive–behavioural self‐leadership strategies such as constructive thought pattern strategies, which emphasise reframing perceptions to enhance self‐regulation and performance [83]. NMs demonstrated an awareness of staff pressures and applied these strategies by adapting their leadership approaches, including planning, coordinating, facilitating, motivating and evaluating to suit their service contexts. Redirecting cognitive and emotional energy away from specific stressors helped build resilience, with NMs viewing themselves as part of larger teams to prioritise and share responsibility [12, 40, 48, 64]. NMs’ personal attributes of being supportive and approachable, maintaining a positive outlook, managing stress effectively and demonstrating strong organisational commitment, further reinforced this cognitive process [44, 57, 81].

###### 4.2.2.1.4. Past Professional Experiences

Four studies (8%) outlined how NMs leveraged past professional experiences to balance dual responsibilities. Coping responses varied according to the extent of experience and employment context [52]. Previously experienced senior roles contributed to the development of leadership capabilities [44], while clinical experience supported NMs’ ability to guide staff and maintain high standards of patient care [38]. NMs drew on these experiences to coordinate programme and service implementation, including identifying and undertaking necessary steps to achieve successful outcomes. This also reinforced their sense of responsibility and accountability within their designated service areas [48].

###### 4.2.2.1.5. Work–Life Balance

Three (6%) studies emphasised maintaining work–life balance was crucial for NMs’ well‐being and effective navigation of role complexities. NMs sought to balance work and home responsibilities without compromising either, aiming to remain fully present and effective in their management roles rather than continually shifting focus between competing demands. Balance, peace and contentment were viewed as essential to their well‐being. Although caring for their physical, emotional and psychological health was recognised as important, NMs struggled to actively pursue strategies to reduce stress and prioritise self‐care [52, 54]. Strategies employed included physical exercise, faith‐based practices, family support and self‐reflection to manage personal demands and cope with professional responsibilities [65]. NMs perceived that investing in personal well‐being reduced stress, improved physical and mental energy and decreased feelings of being overwhelmed [54]. This sense of well‐being enabled them to sustain positive engagement at work, maintain motivation and manage job demands more effectively. It also enhanced their emotional capacity to balance the needs and priorities of leaders and direct reports, maintain realistic expectations and remain flexible in dynamic work environments [54].

##### 4.2.2.2. Subtheme 2: Cognitive‐Focused Strategies

Cognitive‐focused coping strategies reflect structured and problem‐oriented approaches NMs use to manage competing clinical and managerial demands. These strategies involve active problem‐solving, prioritisation and decision‐making processes that support effective role performance in complex and dynamic work environments. This subtheme includes strategies such as planful problem‐solving, deliberate decision‐making strategies, role distancing, leadership presence and accessibility and leadership skills.

###### 4.2.2.2.1. Planful Problem‐Solving

Six (12%) studies reported the use of problem‐solving approaches by NMs to balance dual roles. NMs implemented and refined action plans using analytical thinking, critical reasoning and conflict management skills to support efficient and effective operations, contributing to reduced work‐related fatigue [44, 55, 84]. They adopted strategies such as debriefing problematic situations, deflecting multiple requests, diffusing conflicts, identifying dysfunctional behaviours and regrouping to address emerging issues. These approaches were often combined with proactive engagement with staff to maintain visibility across service areas, foster relationships and provide support, which helped reduce stress and prevent potential issues [12, 40, 64]. As NMs gained experience and progressed beyond initial learning phases, many demonstrated increased confidence and competence in their roles. This included a shift from routine task management to strategic thinking, applying analytical skills to risk management and using structured methods to problem‐solving [52, 57, 80].

###### 4.2.2.2.2. Deliberate Decision‐Making Strategies

Five (10%) studies reported that NMs found it challenging to disengage from work, often continuing work‐related tasks at home, after hours or on weekends to manage competing demands [54, 73]. To address this, they made deliberate decisions about how and when to respond to work demands, supporting prioritisation and more effective role management [65, 73]. This approach aligns with self‐leadership principles, emphasising self‐regulation and reflective decision‐making to enhance performance and well‐being [83]. NMs also reduced meeting attendance to increase presence and accessibility within their service areas [38, 56]. Their decisions informed by situational awareness, past experiences, pattern recognition and self‐reflection [56].

###### 4.2.2.2.3. Role Distancing

One (2%) study identified two distinct orientations among NMs: those aligned with managerial responsibilities and those maintaining strong professional nursing identity [42]. NMs with managerial orientation often distanced themselves from direct clinical responsibilities, maintaining professional connections through organisational activities such as networks, meetings and projects. This was associated with reduced visibility in day‐to‐day operations and patient care discussions, leading to increased delegation of clinical decision‐making to RNs [42]. In contrast, NMs who maintained a professional nursing orientation primarily identified as RNs and deliberately downplayed managerial authority, reinforcing their clinical identity in staff interactions. Although preferring a clinical focus, they remained responsible for administrative tasks and could not fully disengage from managerial duties. As a result, distancing from the management role was less apparent to staff [42].

###### 4.2.2.2.4. Leadership Presence and Accessibility

Six (12%) studies highlighted the importance of proactive leadership, with NMs emphasising visibility and accessibility within service areas to foster relationships, support staff, reduce stress and promote positive work environments [12, 40, 55]. Effective communication was central to maintaining presence and building trust and rapport [40, 55]. NMs also acted as advocates, supporting staff development, strengthening teams and fostering a supportive workplace culture [40, 54, 57]. Establishing clear professional and interpersonal boundaries was identified as essential for managing system‐related pressures and enhancing confidence and effectiveness in the role [74].

###### 4.2.2.2.5. Leadership Skills

Seven (14%) studies highlighted the importance of leadership skills in enabling NMs to balance their dual roles. These skills were reflected in their ability to build, develop and sustain effective multidisciplinary teams that achieved organisational goals. NMs motivated and empowered staff through education and regular in‐service training, while relying on effective communication and collaboration to maintain patient‐centred care [40, 44, 47, 55, 57].

Increased supervisory time was associated with improved time management and the ability to create clinical environments that supported patient and staff safety [40, 43]. Role expectations were more achievable when adequate supervisory time and full staffing capacity were available [44, 47]. Sufficient staffing also enabled delegation of tasks, such as audit data collection, allowing NMs to focus on higher‐level priorities, including fostering supportive work cultures, demonstrating empathy, recognising staff diversity, planning workflows and managing resources. NMs also engaged in direct communication through interviews and feedback to address concerns and support staff performance [47, 55].

Delegation further enhanced team engagement and supported staff development by encouraging participation in managerial functions [40, 44, 47]. As NMs gained experience, they strengthened their leadership and management capabilities, reevaluated their role and developed stronger professional identities. Some also pursued additional skills, including second‐language acquisition, to support broader professional engagement [57].

#### 4.2.3. Theme: 3 Factors Influencing NMs’ Prioritisation and Decision‐Making

NMs constantly navigate competing clinical and managerial demands, necessitating ongoing prioritisation and decision‐making in complex practice environments [11, 70]. Nine (18%) studies identified factors influencing NMs’ prioritisation of clinical and managerial responsibilities. One subtheme emerged within this theme: *Clinical and organisational influences on prioritisation*.

##### 4.2.3.1. Subtheme: Clinical and Organisational Influences on Prioritisation

Clinical and organisational influences on prioritisation highlight factors that shape how NMs allocate time, attention and resources when balancing competing demands. These influences reflect the dynamic interaction between clinical needs, organisational priorities and resource availability in guiding decision‐making. This subtheme includes factors such as clinical presence, staff support, responsiveness to patient and organisational needs, staffing constraints, models of care, role responsibility, professional identity and service‐level work structures. Given the limited and overlapping evidence, these factors are presented collectively.

Five (10%) studies reported that NMs prioritised clinical responsibilities over managerial duties for several reasons [35, 38, 40, 65, 85]. Managing high volume of patient‐related issues was identified as a significant challenge, including responding to concerns from patients and visitors, managing difficult behaviours and addressing high patient acuity, all of which increased nursing workload [40]. These clinical demands, alongside competing organisational and managerial priorities, influenced NMs’ capacity to maintain visibility within clinical settings, potentially affecting patient experience, continuity of care and staff responsiveness [35, 38, 65, 85].

When faced with competing demands, clinical needs were often prioritised despite substantial administrative workloads, resulting in increased time pressure to complete managerial tasks [39, 40]. This prioritisation was driven by the need to maintain a visible clinical presence to support staff, respond to patient and organisational priorities and manage the impact of staff shortages, alongside a strong sense of responsibility for ensuring smooth service delivery [71, 85]. In some cases, maintaining direct patient contact was a deliberate strategy to reinforce their professional identity and mitigate staff frustration associated with organisational policies [35].

Five (10%) studies reported that models of care influenced NMs’ engagement in clinical work, while three (6%) described duty allocation approaches to balance clinical and managerial responsibilities. One study identified an even 50/50 split between leadership and clinical practice [51], whereas another found that NMs spent 27% of their time on nursing services, 27% on human resource management and only 12% on direct patient care, with community‐based NMs allocating more time to human resources than hospital‐based managers [86]. A third study proposed a ten‐question cognitive model to support decision‐making in high‐pressure contexts [80].

Two (4%) studies described three models of role implementation that influenced prioritisation: (i) functioning as an integral member of the clinical team; (ii) adopting a clinically focused but supernumerary role; and (iii) providing support during times of crisis [36, 38]. In the first model, NMs were typically included in nursing roster and maintained strong clinical focus, facilitating accessibility to staff, role modelling of best practice and identification of professional development needs. In the second model, clinical involvement occurred on an ad hoc basis. In the third, NMs were more engaged in operational responsibilities or contributed to clinical practice through advisory roles, such as developing standards and protocols [36, 38]. Overall, clinical engagement was highest when NMs were integrated with frontline staff and lowest when operating in supervisory or support capacities [36, 38].

## 5. Discussion

This scoping review explored how NMs balance clinical and managerial responsibilities within healthcare settings. Forty‐nine studies were included, representing across 13 countries and providing a broad, albeit unevenly distributed, global perspective. Most studies (65%) were conducted in high‐income, English‐speaking countries such as Australia, USA, the United Kingdom, Canada and New Zealand, which share similar healthcare infrastructures and well‐established nursing leadership frameworks. Insights and findings from these studies may be applicable and transferrable to countries with comparable healthcare systems.

An additional 14% of studies originated from Northern and Western Europe, including Finland, Sweden and Norway, where healthcare systems and advanced nursing practice roles are well established. In contrast, only 21% of studies were conducted in non‐Western countries, where healthcare structures and NMs’ roles may differ. The predominance of research from Anglo‐Western countries highlights the need for future studies to capture underrepresented regions and broader international perspectives, thereby informing the development of contextually relevant policies and leadership frameworks.

Of the 49 studies, 39 employed qualitative designs, while five used quantitative methods and five adopted mixed methods approaches. The predominance of qualitative research aligns with the review aim of capturing in‐depth, context‐specific insights into NMs’ experiences, particularly regarding their dual clinical and managerial roles [72, 87, 88]. These studies highlight the complexity of role integration, challenges associated with balancing responsibilities and adaptive strategies employed by NMs. Future research using qualitative approaches could further examine these processes and deepen the understanding of dual‐role complexities. While quantitative studies provide measurable outcomes, their limited representation suggests a gap in capturing nuanced and subjective aspects of role‐balancing among NMs [72, 87, 89]. Mixed methods approaches may offer additional value by integrating contextual depth with broader patterns, thereby providing a more comprehensive understanding of the phenomenon [72, 87, 89].

Semistructured interviews were the most frequently used data collection method, employed in 30 (61%) included studies, reflecting strong emphasis on capturing participants’ personal experiences and perspectives. Other methods included questionnaires, focus groups, direct observation and coaching sessions. Several studies adopted combined methods to enrich data, such as interviews paired with focus groups, questionnaires or direct observation [89–93]. More creative and reflective methods included journal reflections, diary entries, group meetings and dialogue forums [89, 91]. This range of methods highlights the complexity of exploring NMs’ dual roles and underscores the value of methodological diversity in capturing individual insights and contextual dynamics. Within this review, NMs were described using 18 different titles, reflecting considerable variations in role nomenclature across countries and healthcare systems. Despite this diversity, the essence of the role remained consistent, with similar core responsibilities and challenges associated with balancing clinical and managerial demands.

In this review, it was evident that NMs faced an overwhelming array of challenges while attempting to fulfil diverse expectations, demands and responsibilities [1, 12, 36–50, 52–71, 73, 81]. Despite well‐documented pressures, including but not limited to physiological strain, role dissatisfaction, workload overload, burnout and high turnover, NMs continue relentlessly to pursue this leadership role. This apparent paradox raises important questions: What motivates NMs to assume such complex leadership positions? What pathways or processes shape their progression into these roles? What factors influence their decision to remain in this complex leadership role? and Are NMs aware of the role demands and realities of the role before assuming the position? Interestingly, the literature provided limited insights into initial motivations underpinning transitions into the managerial role. However, available evidence suggests that many NMs assumed and remained in the role as they interpreted the role through a lens of professionalism. They perceived the position as an opportunity to uphold nursing standards and exercise professional autonomy, particularly in decision‐making related to resource allocation and patient care [35, 38, 42]. Management was also viewed as a pathway for personal and professional growth, enabling NMs to mentor, supervise and guide staff while reinforcing their professional identity [35, 42]. Progression into NMs’ roles appears influenced by a combination of organisational opportunity, workforce need, professional aspiration and career progression pathways [38, 42, 64].

Another key issue is the common assumption that NMs should manage both clinical and managerial responsibilities. This raises important questions: What justifies combining these roles? What benefits, if any, does this structure provide? And critically, How effective can NMs be when expected to manage multiple responsibilities within a typical workday? Several studies in this review identified that the broad scope of responsibilities makes it difficult for NMs to maintain oversight of all aspects of their roles and perform duties optimally. This often limits their ability to concentrate on specific tasks and results in frequent workflow interruptions and increasing the risk of inattention to detail [41, 48, 56, 63–65]. Therefore, the effectiveness and sustainability of this model warrant further critical evaluation. Future research should explore whether greater role clarity, division or redistribution could enhance both managerial performance and job satisfaction.

This review also raises a reflective question of whether NMs fully understand the scope and intensity of the role before assuming the position. Several studies reported that NMs perceived the role to be more diverse and demanding than outlined in formal job description [40, 75] and highlighted how unprepared they felt upon entering their positions [43, 47, 48, 53, 57, 63, 67, 70, 77]. This significant gap between organisational expectations, as reflected in job descriptions, and the reality of role demands in practice may adversely impact NMs confidence, effectiveness and retention [33, 43, 52, 56]. Consequently, there is a clear need to generate deeper insights and develop theoretical frameworks to support leadership development and role preparedness amongst NMs. Such efforts could better equip healthcare organisations to prepare NMs for their roles, while also prompting education providers to reconsider how nurses are prepared for future management positions.

## 6. Strengths and Limitations

This scoping review employed systematic, transparent and replicable methods throughout and was conducted in alignment with the JBI methodology, following the PRISMA‐ScR checklist [18]. It was prospectively published on the Open Science Framework (Registration DOI: https://doi.org/10.17605/OSF.IO/8CKEM). Included studies were sourced from seven databases and Google Scholar, with no publication date restrictions applied to ensure a comprehensive review of all available evidence. Search terms were refined through discussions with the research team and senior research and learning librarians to enhance precision and identify relevant studies. Selection of studies was guided by inclusion and exclusion criteria developed by a team with expertise in conducting scoping reviews. Covidence supported consistency and accuracy throughout the review, enabling efficient screening of studies, quality appraisal, data extraction and conflict resolution in a collaborative and systematic manner [16, 18, 27]. Quality appraisal allowed for assessment of the overall quality of evidence. Thematic analysis identified recurring patterns, providing a framework to explore relationships between themes and highlight gaps in the literature. These findings offer direction for future research by identifying areas requiring further exploration [33, 34].

While every effort was made to ensure rigour of the review design, several limitations remain. The review included only articles published in English language as the researchers are principally English speaking and due to resource constraints. Therefore, relevant studies in other languages may have been missed. Additionally, although the literature indicated that NMs engaged in multiple, overlapping roles, the review was not designed to explore this broader role complexity in depth, as its primary focus was on how they balance clinical and managerial responsibilities. Inconsistencies in titles and role descriptions across studies may have affected comparability and interpretation of findings. Furthermore, it is possible that relevant studies were not captured due to variations in role titles that reflect similar responsibilities but were not included in the search strategy. Despite these limitations, this review provides a comprehensive representation of complexity of NMs’ roles and offers valuable insights to inform future research, policy development and leadership frameworks aimed at supporting NMs in balancing their responsibilities.

## 7. Conclusions

This scoping review revealed that NMs’ roles are crucial to the success of healthcare organisations. It highlighted the complex, multifaceted and demanding nature of the role, characterised by expectations to manage a broad scope of clinical and managerial responsibilities, meet healthcare delivery standards, ensure patient safety and achieve organisational goals. Despite the challenges that hinder effective role performance, nurses continue to pursue and remain in these positions, often driven by professional values, desire to influence and improve standards of care. However, initial motivations of nurses to assume this role remain unclear, as does the extent to which nurses anticipate the actual demands of the position prior to transition. The dual‐role structure, combining both clinical and managerial responsibilities, raises concerns regarding sustainability and effectiveness. Many NMs report difficulties maintaining oversight across all areas of their role, with frequent workflow interruptions and role overload undermining their capacity to perform effectively.

Despite recognised significance of these roles, existing studies provide limited insights into how NMs navigate and balance competing responsibilities, highlighting a critical gap in understanding the processes that guide their prioritisation and decision‐making. Future research can fill an important gap in existing literature, contributing to the theoretical body of knowledge and informing advancements in nursing leadership, healthcare management and policy development.

## Funding

No funding was received for this scoping review. Open access publishing facilitated by La Trobe University, as part of the Wiley ‐ La Trobe University agreement via the Council of Australasian University Librarians.

## Ethics Statement

Ethical approval was not required for this scoping review, as no primary data were collected. Ethical approval and reporting within the included studies were considered where reported.

## Consent

Informed consent does not apply for this scoping review.

## Conflicts of Interest

The authors declare no conflicts of interest.

## Supporting Information

Additional supporting information can be found online in the Supporting Information section.

## Supporting information


**Supporting Information 1** Table S1. PRISMA‐ScR checklist.

**Supporting Information 2** Table S2. Database search strategy.

**Supporting Information 3** Table S3. Quality appraisal tool.

## Data Availability

The data that supports the findings of this study are available in the supporting information of this article.
