# Supplementary material for: Nurse Managers’ Strategies to Navigate Clinical Leadership and Managerial Responsibilities: A Scoping Review
Source: J Nurs Manag. 2026 Jul 20;2026:2736922. doi: 10.1155/jonm/2736922 (PMC13385200; doi:10.1155/jonm/2736922)
Supplement: Supplementary file 2 — Supporting Information 2 Table S2. Database search strategy. [file JONM-2026-2736922-s002.docx]

**TABLE S2. Database search strategy.**

| **Database** | **Platform** | **Search Date** | **Keywords** |
| --- | --- | --- | --- |
| **Embase**  (n=740) | Ovid | 20 December 2024 | (nurse manager/ OR Nurse Administrator/ OR "nurs* administrator*".mp. OR "nurs*leader*".mp. OR "nurs* supervisor*".mp. OR "nurs* coordinator*".mp. OR “nurs* unit manager*".mp. OR "nurs* manager*".mp. OR ward manager*.mp. OR ward sister*.mp. OR nurs* sister*.mp.) AND ((clinical care OR clinical responsibilit* OR clinical dut* OR clinical role* OR clinical function* OR clinical leadership).mp. OR (manag* role* OR manag* dut* OR manag* function* OR manag* responsibilit*).mp. OR (dual role* OR dual responsibilit* OR dual function* OR dual dut*).mp. OR (hybrid role* OR hybrid nurs* manager* OR hybrid responsibilit* OR hybrid nurs* unit manager* OR hybrid function* OR hybrid dut*).mp.) |
| **CINAHL**  (n=762) | EBSCO | 26 December 2024 | ((MH "Nurse Administrators") OR "nurs* manager*” OR "nurs* administrator*" OR "nurs* leader*" OR "nurs* supervisor*" OR "nurs* coordinator*" OR "nurs* unit manager*") AND ("clinical dut*" OR "clinical leadership" OR "clinical function*" OR "clinical care" OR "clinical responsibilit*" OR "clinical role*" OR "manag* dut*" OR manag* responsibilit*" OR "manag* function*" OR "manag* role*" OR "dual* role*" OR "dual responsibilit*" OR "dual function*" OR "dual dut*" OR "hybrid role*" OR "hybrid nurs* manager*" OR "hybrid responsibilit*" OR "hybrid nurs* unit manager*" OR "hybrid function*" OR "hybrid dut*") |
| **Medline**  (n=751) | Ovid | 26 December 2024 | (Nurse Administrators/ OR "nurs* administrator*".mp. OR "nurs* leader*".mp. OR "nurs* supervisor*".mp. OR "nurs* coordinator*".mp. OR "nurs* unit manager*".mp. OR "nurs* manager*".mp. OR ward manager*.mp. OR ward sister*.mp. OR nurs* sister*.mp.) AND ((clinical care OR clinical responsibilit* OR clinical dut* OR clinical role* OR clinical function* OR clinical leadership).mp. OR (manag* role* OR manag* dut* OR manag* function* OR manag* responsibilit*).mp. OR (dual role* OR dual responsibilit* OR dual function* OR dual dut*).mp.    OR (hybrid role* OR hybrid nurs* manager* OR hybrid responsibilit* OR hybrid nurs* unit manager* OR hybrid function* OR hybrid dut*).mp.) |
| **ProQuest Central**  (n=5999) | ProQuest | 26 December 2024 | (noft (nurs* administrator* OR nurs* leader* OR nurs* supervisor* OR nurs* coordinator* OR nurs* unit manager* OR nurs* manager* OR ward manager* OR ward sister* OR nurs* sister*) AND (noft(clinical care OR clinical responsibilit* OR clinical dut* OR clinical role* OR clinical function* OR clinical leadership) OR noft(manag* role* OR manag* dut* OR manag* function* OR manag* responsibilit*) OR noft(dual role* OR dual responsibilit* OR dual function* OR dual dut*) OR noft(hybrid role* OR hybrid nurs* manager* OR hybrid responsibilit* OR hybrid nurs* unit manager* OR hybrid function* OR hybrid dut*))) NOT at.exact("Obituary" OR "Speech/Lecture" OR "Correction/Retraction" OR "Legal Materials" OR "Credit/Acknowledgement" OR "Recipe") AND la.exact("ENG") AND PEER(yes) NOT subt.exact( "medical personnel" OR "physicians" OR "clinical medicine" OR "students" OR "emergency medical care" OR "medical research" OR "occupational health" OR "medicine" OR "surgery" OR "clinical trials" OR "content analysis" OR "coronaviruses" OR "data analysis" OR "dementia" OR "quality control" OR "womens health") |
| **PubMed**  (n=4415) | PubMed | 26 December 2024 | (("Nurse Administrators"[Mesh:NoExp]) OR (nurs* administrator*) OR (nurs* coordinator*) OR (nurs* leader) OR (nurs* sister) OR (ward sister*) OR (ward manager*) OR (nurs* supervisor*) OR (nurs* unit manager) OR (nurs* manager)) AND (("clinical care") OR ("clinical responsibilit*") OR ("clinical dut*") OR ("clinical role*") OR ("clinical function*") or ("clinical leadership") OR ("manag* role*") OR ("manag* dut*") OR ("manag* function*") OR ("manag* responsibilit*") OR ("dual role*") OR ("dual responsibilit*") OR ("dual function*") OR ("dual dut*") OR ("hybrid role*") OR ("hybrid nurs* manager*") OR ("hybrid responsibility"[tiab:~0]) OR ("hybrid responsibilities"[tiab:~0]) OR ("hybrid managerial responsibilities"[tiab:~0]) OR ("hybrid managerial responsibility"[tiab:~0]) OR ("hybrid nurse unit manager"[tiab:~0]) OR ("hybrid nurse unit managers"[tiab:~0]) OR ("hybrid nursing unit manager"[tiab:~0]) OR ("hybrid nursing unit managers"[tiab:~0]) OR ("hybrid function*") OR ("hybrid duty"[tiab:~0]) OR ("hybrid duties"[tiab:~0])) |
| **Scopus**  (n=874) | Elsevier | 26 December 2024 | (TITLE-ABS-KEY("nurs* administrator*") OR TITLE-ABS-KEY("nurs* leader*") OR TITLE-ABS-KEY("nurs* supervisor*") OR TITLE-ABS-KEY("nurs* coordinator*") OR TITLE-ABS-KEY("nurs* unit manager*") OR TITLE-ABS-KEY("nurs* manager*") OR TITLE-ABS-KEY("ward manager*")    OR TITLE-ABS-KEY ("ward sister*") OR TITLE-ABS-KEY ("nurs* sister*")) AND (TITLE-ABS-KEY ("clinical dut*") OR TITLE-ABS-KEY ("clinical care") OR TITLE-ABS-KEY ("clinical responsibilit*") OR TITLE-ABS-KEY ("clinical role*") OR TITLE-ABS-KEY ("clinical function*") OR TITLE-ABS-KEY("clinical leadership*") OR TITLE-ABS-KEY("manag* dut*") OR TITLE-ABS-KEY("manag* role*") OR TITLE-ABS-KEY("manag* function*") OR TITLE-ABS-KEY("manag* responsibilit*") OR TITLE-ABS-KEY("dual* role*") OR TITLE-ABS-KEY("dual* responsibilit*") OR TITLE-ABS-KEY("dual* function*") OR TITLE-ABS-KEY("dual* dut*") OR TITLE-ABS-KEY("hybrid role*") OR TITLE-ABS-KEY("hybrid nurs* manager") OR TITLE-ABS-KEY("hybrid responsibilit*") OR TITLE-ABS-KEY ("hybrid nurs unit manager*") OR TITLE-ABS-KEY ("hybrid dut*") OR TITLE-ABS-KEY ("hybrid function*")) |
| **Web of Science**  (n=443) | Clarivate | 26 December 2024 | ALL=(( "nurs* sister*" OR "ward sister*" OR "ward manager*" OR "nurs* manager*" OR "nurs* unit manager*" OR "nurs* coordinator*" OR "nurs* supervisor*" OR "nurs* leader*" OR "nurs* administrator*") AND ("clinical care" OR "clinical responsibilit*" OR "clinical dut*" OR "clinical role*" OR "clinical function*" OR "clinical leadership" OR "manag* role*" OR "manag* dut*" OR "manag* function*" OR "manag* responsibilit*" OR "dual role*" OR "dual responsibilit*" OR "dual function*" OR "dual dut*" OR "hybrid role*" OR "hybrid nurs* manager*" OR "hybrid responsibilit*" OR "hybrid nurs* unit manager*" OR "hybrid function*" OR "hybrid dut*")) |
