# Supplementary material for: Nurse Managers’ Strategies to Navigate Clinical Leadership and Managerial Responsibilities: A Scoping Review
Source: J Nurs Manag. 2026 Jul 20;2026:2736922. doi: 10.1155/jonm/2736922 (PMC13385200; doi:10.1155/jonm/2736922)
Supplement: Supplementary file 3 — Supporting Information 3 Table S3. Quality appraisal tool. [file JONM-2026-2736922-s003.docx]

**TABLE S3. Quality appraisal table using CASP tools.**

| Paper No. | Author/s Year | Study Title | Study Design | Type of CASP Tool | Q1 | Q2 | Q3 | Q4 | Q5 | Q6 | Q7 | Q8 | Q9 | Q10 | Q11 | Q12 | SS | RC | Overall Quality Judgment  Score | Notes  Comments |
| --- | --- | --- | --- | --- | --- | --- | --- | --- | --- | --- | --- | --- | --- | --- | --- | --- | --- | --- | --- | --- |
| 1 | Algunmeeyn et al. (2023) | Effective clinical nursing leadership in hospitals: Barriers from the perspectives of nurse managers | Qualitative | CASP Qualitative | Y  Y | Y  Y | Y  Y | Y  Y | Y  Y | Y  Y | Y  Y | Y  Y | Y  Y | Y  Y | -  - | -  - | 10 | 10 | **10/10**  **High**  **100%** |  |
| 2 | Andersson et al. (2014) | Management of everyday work in Emergency Departments - an exploratory study with Swedish Managers | Qualitative | CASP Qualitative | Y  Y | Y  Y | Y  Y | Y  Y | Y  Y | N  N | Y  Y | Y  Y | Y  Y | Y  Y | -  - | -  - | 9 | 9 | **9/10**  **High**  **90%** |  |
| 3 | Bolton (2003) | Multiple roles? Nurses as managers in the NHS | Qualitative | CASP Qualitative | Y  Y | Y  Y | Y  Y | Y  CT | N  N | N  N | N  N | CT  CT | Y  Y | Y  Y | -  - | -  - | 5 | 6 | **5.5/10**  **Moderate**  **55%** | Methodology, including number of participants, not well described.  *Since this article is part of a larger longitudinal study, some questions in the Appraisal Tool may have already been addressed in the initial publication. |
| 4 | Bunsey et al. (1991) | Nurse managers: role expectations and job satisfaction | Cross-sectional | CASP Cross-Sectional | Y  Y | Y  Y | CT  Y | Y  Y | Y  Y | CT  CT | Y  Y | Y  Y | Y  Y | Y  Y | Y  Y | -  - | 10 | 9 | **9.5/11**  **High**  **86%** |  |
| 5 | Chisengantambu-Winters et al. (2024) | Exploring the multifaceted and multifunctional roles and activities of rural nurse managers | Qualitative | CASP Qualitative | Y  Y | Y  Y | Y  Y | Y  Y | Y  Y | N  N | Y  Y | Y  Y | Y  Y | Y  Y | -  - | -  - | 9 | 9 | **9/10**  **High**  **90%** |  |
| 6 | Cilliers & Terblanche (2010) | The systems psychodynamic leadership coaching experiences of nursing managers | Qualitative | CASP Qualitative | Y  Y | Y  Y | Y  Y | Y  Y | Y  Y | Y  N | Y  Y | Y  Y | Y  Y | Y  Y | -  - | -  - | 10 | 9 | **9.5/10**  **High**  **95%** |  |
| 7 | Cziraki et al. (2014) | Factors that facilitate registered nurses in their first-line nurse manager role | Qualitative | CASP Qualitative | Y  Y | Y  Y | Y  Y | Y  Y | Y  Y | N  N | Y  Y | Y  Y | Y  Y | Y  Y | -  - | -  - | 9 | 9 | **9/10**  **High**  **90%** |  |
| 8 | Dienemann & Shaffer (1992) | Manager responsibilities in community agencies and hospitals | Mixed methods | CASP Qualitative & | Y  Y | Y  Y | Y  Y | Y  Y | Y  Y | N  N | N  N | Y  Y | Y  Y | Y  Y | -  - | -  - | 8 | 8 | **8/10**  **High (80%)**  **8/12 (67%)**  **Moderate** | **73.5%** |
|  |  |  |  | CASP Cohort | Y  Y | Y  Y | Y  Y | Y  Y | CT  CT | N  N | Y  Y | Y  Y | Y  Y | Y  Y | Y  Y | Y  Y | 8 | 8 |  |  |
| 9 | Drach-Zahavy & Dagan (2002) | From caring to managing and beyond: an examination of the head nurse's role | Qualitative - observational | CASP Qualitative | Y  Y | Y  Y | Y  Y | N  N | Y  Y | N  N | N  N | Y  Y | Y  Y | Y  Y | -  - | -  - | 7 | 7 | **7/10**  **Moderate**  **70%** | Potential for observer bias. |
| 10 | Duffield et al. (2019) | Manager, clinician or both? Nurse managers' engagement in clinical care activities | Cross-sectional | CASP Cross-Sectional | Y  Y | Y  Y | Y  Y | Y  Y | Y  Y | Y  Y | Y  Y | Y  Y | Y  Y | Y  Y | Y  Y | -  - | 11 | 11 | **11/11**  **High**  **100%** |  |
| 11 | El Haddad et al. (2022) | Nurse unit managers' work and impacts on clinical leadership: A cross-sectional study | Cross-sectional | CASP Cross-Sectional | Y  Y | Y  Y | Y  Y | CTCT | Y  Y | Y  Y | Y  Y | Y  Y | Y  Y | Y  Y | Y  Y | -  - | 11 | 11 | **11/11**  **High**  **100%** |  |
| 12 | Ericsson & Augustinsson (2015) | The role of first line managers in healthcare organisations: a qualitative study on the work life experience of ward managers | Multiple methods (all qualitative) | CASP Qualitative | Y  Y | Y  Y | Y  Y | Y  Y | Y  Y | N  N | N  N | Y  Y | Y  Y | Y  Y | -  - | -  - | 8 | 8 | **8/10**  **High**  **80%** | Approval from ethics committee not stated |
| 13 | Gaskin et al. (2012) | The challenges acute care nurse unit managers face and the strategies they use to address them: Perceptions of directors of nursing and nurse unit managers | Qualitative | CASP Qualitative | Y  Y | Y  Y | Y  Y | Y  Y | Y  Y | N  N | Y  Y | Y  Y | Y  Y | Y  Y | -  - | -  - | 9 | 9 | **9/10**  **High**  **90%** |  |
| 14 | Gould (2008) | The matron's role in acute National Health Service trusts | Qualitative | CASP Qualitative | Y  Y | Y  Y | Y  Y | Y  Y | Y  Y | N  N | Y  Y | Y  Y | Y  Y | Y  Y | -  - | -  - | 9 | 9 | **9/10**  **High**  **90%** |  |
| 15 | Ingersoll et al. (1999) | The effect of patient-focused redesign on midlevel nurse managers' role responsibilities and work environment | Qualitative | CASP Qualitative | Y  Y | Y  Y | Y  Y | Y  Y | Y  Y | N  N | N  CT | Y  Y | Y  Y | Y  Y | -  - | -  - | 8 | 8 | **8/10**  **High**  **80%** | Approval from ethics committee not stated |
| 16 | Kagan et al. (2021) | A mixed-methods study of nurse managers' managerial and clinical challenges in mental health centres during the COVID-19 pandemic | Mixed methods | CASP Qualitative & | Y  Y | Y  Y | Y  Y | Y  Y | Y  Y | N  N | Y  Y | Y  Y | Y  Y | Y  Y | -  - | -  - | 9 | 9 | **9/10**  **High (90%)**  **8/12**  **Moderate**  **(67%)** | **78.5%** |
|  |  |  |  | CASP Cohort | Y  Y | CT  CT | CTCT | CTCT | Y  Y | CTCT | Y  Y | Y  Y | Y  Y | Y  Y | Y  Y | Y  Y | 8 | 8 |  |  |
| 17 | Keutchafo & Kerr (2019) | Difficulties of unit managers in selected district hospitals in Cameroon | Qualitative | CASP Qualitative | Y  Y | Y  Y | Y  Y | Y  Y | Y  Y | Y  Y | Y  Y | Y  Y | Y  Y | Y  Y | -  - | -  - | 10 | 10 | **10/10**  **High**  **100%** |  |
| 18 | Kirchhoff & Karlsson (2018) | Alternative careers at the first level of management: first-line nurse managers' responses to role conflict | Qualitative | CASP Qualitative | Y  Y | Y  Y | Y  Y | Y  Y | Y  Y | N  N | Y  Y | Y  Y | Y  Y | Y  Y | -  - | -  - | 9 | 9 | **9/10**  **High**  **90%** | Small sample size, data saturation not achieved. |
| 19 | Luo et al. (2016) | Exploring competencies: a qualitative study of Chinese nurse managers | Qualitative | CASP Qualitative | Y  Y | Y  Y | Y  Y | Y  Y | Y  Y | N  N | Y  Y | Y  Y | Y  Y | Y  Y | -  - | -  - | 9 | 9 | **9/10**  **High**  **90%** |  |
| 20 | Maguire et al. (2023) | Exploring the role of the nurse unit manager in forensic mental health inpatient units: A qualitative study | Qualitative | CASP Qualitative | Y  Y | Y  Y | Y  Y | Y  Y | Y  Y | Y  Y | Y  Y | Y  Y | Y  Y | Y  Y | -  - | -  - | 10 | 10 | **10/10**  **High**  **100%** |  |
| 21 | McCallin & Frankson (2010) | The role of the charge nurse manager: a descriptive exploratory study | Qualitative | CASP Qualitative | Y  Y | Y  Y | Y  Y | Y  Y | Y  Y | N  N | Y  Y | Y  Y | Y  Y | Y  Y | -  - | -  - | 9 | 9 | **9/10**  **High**  **90%** |  |
| 22 | McEwen et al. (2005) | Are ward sisters and charge nurses able to fulfil their role? | Cross-sectional | CASP Cross-Sectional | Y  Y | Y  Y | Y  Y | CT  CT | Y  Y | CT  CT | Y  Y | CTCT | Y  Y | Y  Y | Y  Y | -  - | 8 | 8 | **8/11**  **Moderate**  **73%** |  |
| 23 | Moore et al. (2016) | Nurse managers' insights regarding their role highlight the need for practice changes | Qualitative | CASP Qualitative | Y  Y | Y  Y | Y  Y | Y  Y | Y  Y | Y  Y | Y  Y | Y  Y | Y  Y | Y  Y | -  - | -  - | 10 | 10 | **10/10**  **High**  **100%** |  |
| 24 | Nagle et al. (2021) | Valuing nurse and midwifery unit managers' voices: a qualitative approach | Qualitative | CASP Qualitative | Y  Y | Y  Y | Y  Y | Y  Y | Y  Y | N  N | Y  Y | Y  Y | Y  Y | Y  Y | -  - | -  - | 9 | 9 | **9/10**  **High**  **90%** |  |
| 25 | Narinen & Kekki (2003) | The content of the nurse manager's work in Finland | Cross-sectional | CASP Cross-Sectional | Y  Y | Y  Y | Y  Y | Y  Y | Y  Y | N  N | Y  Y | Y  Y | Y  Y | Y  Y | Y  Y | -  - | 10 | 10 | **10/11**  **High**  **91%** |  |
| 26 | Nene (2022) | Nurse managers' leadership roles in mining primary healthcare settings in Gauteng | Qualitative | CASP Qualitative | Y  Y | Y  Y | Y  Y | Y  Y | Y  Y | Y  Y | Y  Y | Y  Y | Y  Y | Y  Y | -  - | -  - | 10 | 10 | **10/10**  **High**  **100%** |  |
| 27 | Nene et al. (2020) | Nurse managers experiences of their leadership roles in a specific mining primary healthcare service in the West Rand | Qualitative | CASP Qualitative | Y  Y | Y  Y | Y  Y | Y  Y | Y  Y | Y  Y | Y  Y | Y  Y | Y  Y | Y  Y | -  - | -  - | 10 | 10 | **10/10**  **High**  **100%** |  |
| 28 | Nurmeksela et al. (2021) | Relationships between nurse managers’ work activities, nurses’ job satisfaction, patient satisfaction, and medication errors at the unit level: a correlational study | Cross-sectional | CASP Cross-Sectional | Y  Y | Y  Y | Y  Y | Y  Y | Y  Y | Y  Y | Y  Y | Y  Y | Y  Y | Y  Y | Y  Y | -  - | 11 | 11 | **11/11**  **High**  **100%** |  |
| 29 | Paliadelis (2008) | The working world of nursing unit managers: responsibility without power | Qualitative | CASP Qualitative | Y  Y | Y  Y | Y  Y | Y  Y | Y  Y | Y  Y | Y  Y | Y  Y | Y  Y | Y  Y | -  - | -  - | 10 | 10 | **10/10**  **High**  **100%** |  |
| 30 | Paliadelis (2005) | Rural nursing unit managers: education and support for the role | Qualitative | CASP Qualitative | Y  Y | Y  Y | Y  Y | Y  Y | Y  Y | Y  Y | Y  Y | Y  Y | Y  Y | Y  Y | -  - | -  - | 10 | 10 | **10/10**  **High**  **100%** |  |
| 31 | Pegram et al. (2015) | An exploration of the working life and role of the ward manager within an acute care hospital organisation | Cross-sectional | CASP Cross-Sectional | Y  Y | Y  Y | Y  Y | Y  Y | Y  Y | Y  Y | Y  Y | Y  Y | Y  Y | Y  Y | Y  Y | -  - | 11 | 11 | **11/11**  **High**  **100%** |  |
| 32 | Rankin et al. (2016) | Facilitators and barriers to the increased supervisory role of senior charge nurses: a qualitative study | Mixed methods | CASP Qualitative & | Y  Y | Y  Y | Y  Y | Y  Y | Y  Y | N  N | Y  Y | Y  Y | Y  Y | Y  Y | -  - | -  - | 9 | 9 | **9/10**  **High (90%)**  **10/12**  **High (83%)** |  |
|  |  |  |  | CASP Cohort | Y  Y | Y  Y | Y  Y | Y  Y | N  N | CTCT | Y  Y | Y  Y | Y  Y | Y  Y | Y  Y | Y  Y | 10 | 10 |  |  |
| 33 | Savage & Scott (2004) | The modern matron: a hybrid management role with implications for continuous quality improvement | Multiple methods (all qualitative) | CASP Qualitative | Y  Y | Y  Y | Y  Y | CTCT | Y  Y | N  N | N  N | Y  Y | Y  Y | Y  Y | -  - | -  - | 7 | 7 | **7/10**  **Moderate**  **70%** |  |
| 34 | Scott & Timmons (2017) | Tensions within management roles in healthcare organisations | Qualitative | CASP Qualitative | Y  Y | Y  Y | Y  Y | Y  Y | Y  Y | Y  Y | Y  Y | Y  Y | Y  Y | Y  Y | -  - | -  - | 10 | 10 | **10/10**  **High**  **100%** |  |
| 35 | Shirey (2009) | Stress and coping in nurse managers: a qualitative description | Qualitative | CASP Qualitative | Y  Y | Y  Y | Y  Y | Y  Y | Y  Y | Y  Y | Y  Y | Y  Y | Y  Y | Y  Y | -  - | -  - | 10 | 10 | **10/10**  **High**  **100%** |  |
| 36 | Shirey et al. (2010) | Understanding nurse manager stress and work complexity: factors that make a difference | Qualitative | CASP Qualitative | Y  Y | Y  Y | Y  Y | Y  Y | Y  Y | N  N | Y  Y | Y  Y | Y  Y | Y  Y | -  - | -  - | 9 | 9 | **9/10**  **High**  **90%** |  |
| 37 | Shirey et al. (2013) | Nurse manager cognitive decision-making amidst stress and work complexity | Qualitative | CASP Qualitative | Y  Y | Y  Y | Y  Y | Y  Y | Y  Y | N  N | Y  Y | Y  Y | Y  Y | Y  Y | -  - | -  - | 9 | 9 | **9/10**  **High**  **90%** |  |
| 38 | Smith et al. (2024) | Exploring presence practices: a study of unit managers in a selected Provincial Hospital in Free State Province | Qualitative | CASP Qualitative | Y  Y | Y  Y | Y  Y | Y  Y | Y  Y | Y  Y | Y  Y | Y  Y | Y  Y | Y  Y | -  - | -  - | 10 | 10 | **10/10**  **High**  **100%** |  |
| 39 | Surakka (2008) | The nurse manager's work in the hospital environment during the 1990s and 2000s: responsibility, accountability and expertise in nursing leadership | Qualitative | CASP Qualitative | Y  Y | Y  Y | Y  Y | CT  CT | Y  Y | N  N | CT  CT | Y  Y | Y  Y | Y  Y | -  - | -  - | 7 | 7 | **7/10**  **Moderate**  **70%** |  |
| 40 | Taylor et al. (2015) | Nurse managers' strategies for feeling less drained by their work: an action research and reflection project for developing emotional intelligence | Qualitative – Action research | CASP Qualitative | Y  Y | Y  Y | Y  Y | Y  Y | Y  Y | N  N | Y  Y | Y  Y | Y  Y | Y  Y | -  - | -  - | 9 | 9 | **9/10**  **High**  **90%** |  |
| 41 | Townsend et al. (2015) | Opening the black box in nursing work and management practice: the role of ward managers | Qualitative | CASP Qualitative | Y  Y | Y  Y | Y  Y | Y  Y | Y  Y | N  N | Y  Y | Y  Y | Y  Y | Y  Y | -  - | -  - | 9 | 9 | **9/10**  **High**  **90%** |  |
| 42 | Udod & Care (2011) | Nurse managers’ work stressors and coping experiences: unravelling the evidence | Qualitative | CASP Qualitative | Y  Y | Y  Y | Y  Y | Y  Y | Y  Y | N  N | Y  Y | Y  Y | Y  Y | Y  Y | -  - | -  - | 9 | 9 | **9/10**  **High**  **90%** |  |
| 43 | Udod & Care (2013) | Walking a tight rope: an investigation of nurse managers’ work stressors and coping experiences | Qualitative | CASP Qualitative | Y  Y | Y  Y | Y  Y | Y  Y | Y  Y | Y  Y | Y  Y | Y  Y | Y  Y | Y  Y | -  - | -  - | 10 | 10 | **10/10**  **High**  **100%** |  |
| 44 | Udod et al. (2017a) | Role stressors and coping strategies among nurse managers | Qualitative | CASP Qualitative | Y  y | Y  Y | Y  Y | Y  Y | Y  Y | N  N | Y  Y | Y  Y | Y  Y | Y  Y | -  - | -  - | 9 | 9 | **9/10**  **High**  **90%** |  |
| 45 | Udod et al. (2017b) | Impact of Role Stressors on the Health of Nurse Managers | Qualitative | CASP Qualitative | Y  Y | Y  Y | Y  Y | Y  Y | Y  Y | N  N | Y  Y | Y  Y | Y  Y | Y  Y | -  - | -  - | 9 | 9 | **9/10**  **High**  **90%** |  |
| 46 | Urban et al. (2023) | Acute care nurse managers' definitions of and barriers to well-being: A thematic analysis of open-ended survey questions | Qualitative | CASP Qualitative | Y  Y | Y  Y | Y  Y | Y  Y | Y  Y | N  N | Y  Y | Y  Y | Y  Y | Y  Y | -  - | -  - | 9 | 9 | **9/10**  **High**  **90%** |  |
| 47 | Urquhart et al. (2018) | Making It Happen: Middle Managers' Roles in Innovation Implementation in Health Care | Qualitative | CASP Qualitative | Y  Y | Y  Y | Y  Y | Y  Y | Y  Y | N  N | Y  Y | Y  Y | Y  Y | Y  Y | -  - | -  - | 9 | 9 | **9/10**  **High**  **90%** |  |
| 48 | Vasset et al. (2023) | Nurse leaders' changing roles over 25 years: a qualitative study | Qualitative | CASP Qualitative | Y  Y | Y  Y | Y  Y | Y  Y | Y  Y | N  N | Y  Y | Y  Y | Y  Y | Y  Y | -  - | -  - | 9 | 9 | **9/10**  **High**  **90%** |  |
| 49 | Wong (1998) | The nurse manager as a professional-managerial class: a case study | Qualitative - Case study | CASP Qualitative | Y  Y | Y  Y | Y  Y | Y  Y | Y  Y | N  N | N  N | Y  Y | Y  Y | Y  Y | -  - | -  - | 8 | 8 | **8/10**  **High**  **80%** |  |

**Column Descriptions:**

- **Paper No.**: Number your papers 1–49.
- **Author(s), Year**: Main authors and publication year.
- **Study Title**: Title of the study.
- **Study Design**: E.g., Qualitative, Cross-sectional, Case Study, etc.
- **CASP Tool Used**: Specify which CASP checklist was applied.
- **Q1–Q11**: Each corresponds to the numbered questions in the relevant CASP checklist. Use Y (Yes), N (No), CT (Can't tell).
- **Note**: Not all tools have 10 questions; leave extras blank or mark with "—" if not applicable.
- **Overall Quality Judgment**: based on the appraisal.

**High** (8-10), **Moderate** (4-7), **Low** (1-3) **OR**

**High** (10-12), **Moderate** (5-8), **Low** (1-4) **OR**

**High** (9-11), **Moderate** (5-8), **Low** (1-4)

- **Notes/Comments**: Brief justification or key insights.
